# Soil health and microbial diversity across land-use types: Evidence for agroecological management in peri-urban areas

**DOI:** 10.12688/openreseurope.22066.2

**Published:** 2026-05-26

**Authors:** Caterina A.M. La Porta, Edoardo Marchi, Gemma Chiaffarelli, Ilda Vagge, Valentina Vaglia, Pietro De Marinis, Stefano Zapperi, Stefano Bocchi

**Affiliations:** 1 Department of Environmental Science and Policy, University of Milan, via Celoria 10, Milano, 20133, Italy; 2 Center for Complexity and Biosystems, University of Milan, Milano, 20133, Italy; 3 Fondazione IRCCS Ca' Granda Ospedale Maggiore Policlinico, via Francesco Storza 28, Milano,, 20122, Italy; 4 Departiment of Agricoltural and environmental science, University of Milan, Milano, 20133, Italy; 5 Department of Earth and Environmental Science, University of Pavia, Pavia, 27100, Italy; 6 Department of Physics, University of Milan, via Celoria 16, Milano, 20133, Italy; 7 Istituto di Chimica della Materia Condensata e di Tecnologie per l'Energia Consiglio Nazionale delle Ricerche, Milano, 20125, Italy

**Keywords:** soil microbiome, agroecological management, soil health

## Abstract

**Background:**

Land-use change profoundly influences soil microbial communities, yet its impacts on richness and diversity remain incompletely resolved across taxa.

**Methods:**

Here, we characterized fungal and bacterial communities in soils from four contrasting land-use types—such as crop, reforested, agroforestry, and uncultivated land—located in the same pedoclimatic conditions, using high-throughput amplicon sequencing of Internal Transcribed Spacer (ITS) and 16S ribosomal RNA (rRNA) genes. We quantified species richness and Shannon diversity and examined their relationships with key physicochemical parameters.

**Results:**

Our results reveal that fungal and bacterial communities responded differently to land-use management. Fungal richness was highest in reforested soils, whereas bacterial richness was more uniformly distributed across land uses. Shannon diversity showed greater sensitivity than richness, indicating strong effects of evenness in structuring communities. Multivariate ordinations and correlation analyses further demonstrated that soil properties such as pH, total nitrogen, and cation exchange capacity were significant drivers of microbial community composition and diversity patterns.

**Conclusions:**

Our study provides mechanistic insight into how land management shapes biodiversity and informs strategies for enhancing soil health and ecosystem resilience.

## Plain language summary

Healthy soils support food production, clean water, and climate resilience. In this study, we examined how different land uses—crop fields, agroforestry, uncultivated areas, and reforestation—affect soil health in a peri-urban area of Milan. We analysed soil microbes, which are essential for nutrient cycling and ecosystem stability, along with key soil properties such as pH, nitrogen, and organic matter.

We found that land use strongly shapes microbial communities. Agroforestry soils hosted the highest number of unique microbial species, highlighting their ecological value, while reforested soils showed lower richness due to their early successional stage. Fungal communities were more sensitive to land management than bacterial ones. Soil chemical properties, especially cation-exchange capacity and nitrogen, played a major role in determining microbial patterns.

Overall, our results show that agroforestry can enhance soil biodiversity and resilience, supporting its role as a nature-based solution for sustainable land management in peri-urban landscapes.

## Introduction

For decades, conventional agricultural management has led to a gradual impoverishment and simplification of agricultural landscapes, compromising the overall health of the most productive areas. ^1^ Since the 1950s, agricultural landscapes have been progressively stripped of the ecological infrastructure that for centuries had supported their ecological, productive, and cultural functions. ^2^ This has led to the disruption of the multiple relationships and self-regulation mechanisms within the agroecosystem. As a result, the system has become vulnerable and unstable, highly dependent on external inputs to sustain its productive function, ^3^ unable to support other ecosystem functions across different scales – from soil domain, to the individual field, to the farming system, to the local landscape context and, more broadly, to the landscape unit. ^4^

When considering the subset of peri-urban agricultural systems - ecotonal zones where the thinning of the urban fabric gradually gives way to natural, forestry, and pastoral land uses - these processes are further exacerbated. While urban systems confer numerous benefits to their inhabitants, they are also responsible for the profound alteration of the functioning of local and global ecosystems. This is achieved by the fragmentation and degradation of agricultural and natural habitats, leading to a reduction in biodiversity. Furthermore, urban systems disrupt hydrological systems, and modify energy flows and nutrient cycles. These dynamics, together with unsustainable land-use practices, affect agroecosystem functions and the provision of key ecosystem services (ES) on which the very livelihood of urban populations depends, e.g. supply of clean water, maintenance of soil fertility and health, food production, climate regulation and climate change mitigation, improvement of air quality. ^5^ Nonetheless, peri-urban fringe areas hold considerable potential for providing buffering and resilience functions for cities. ^6^ ^–^ ^11^

Soil health - commonly defined as soil’s ability to function as an ecosystem that sustains plants, animals, and human life - is increasingly recognized as a cornerstone of the broader concept of Global Health, as it underpins agricultural productivity, ecosystem services, human nutrition, and climate resilience. This is particularly relevant in peri-urban areas, where soils are expected to sustain food production while being exposed to intense pressures from urbanization and land-use change. This complexity requires the development of composite indicators that are rapid, sensitive, and reliable in capturing the multifunctionality of soils. Many recent papers explicitly call for harmonized methodologies able to link soil health assessment with food security, biodiversity, and climate policies. ^12^ ^–^ ^15^ In this perspective, soil health indicators should not only measure physical and chemical properties, but also integrate biological and functional dimensions, thereby providing a comprehensive and policy-relevant assessment tool.

To address these challenges, it is therefore necessary to rethink the territorial development of peri-urban fringes in order to maximize their delivering capacity of ES, enhancing their capacity to mitigate the negative externalities of urban systems. Recent advances in the literature highlight the potential of silvoarable agroforestry systems (SAFs) as a strategy to counteract soil degradation and biodiversity loss in Western Europe. According to De Clercq et al., ^16^ SAFs contribute to soil biological health by fostering richer and more diverse soil communities and improving soil organic matter, litter-feeding macrofauna, and arbuscular mycorrhizal fungi, while also reducing soil bulk density. These effects are most pronounced in older stands and in proximity to tree rows, underscoring the role of temporal and spatial factors in shaping belowground processes. Despite these benefits, important research gaps remain—particularly concerning mesofauna, microbial activity, young stands, and deeper soil layers—indicating the need for more integrative indices that combine multiple biological parameters. Incorporating such findings into the broader context of sustainable land management underscores SAFs as a promising practice to enhance ecosystem services and soil resilience under climate and agricultural pressures.

In this context, in the present study we investigated soil health of different land uses representative of typical northern Italy peri-urban agricultural landscapes, i.e field annual crops, agroforestry, wooded areas, set-aside fields by integrating a variety of indicators such as (i) soil physicochemical properties linked to fertility and structure, (ii) microbial community composition and alpha-diversity derived from 16S ITS amplicon sequencing, (iii) multivariate analyses to relate community structure to environmental gradients (PCA/RDA), and (iv) prediction of functional potential of bacterial communities using PICRUSt2.

## Results

### Community composition and diversity patterns

**Figure 1.**
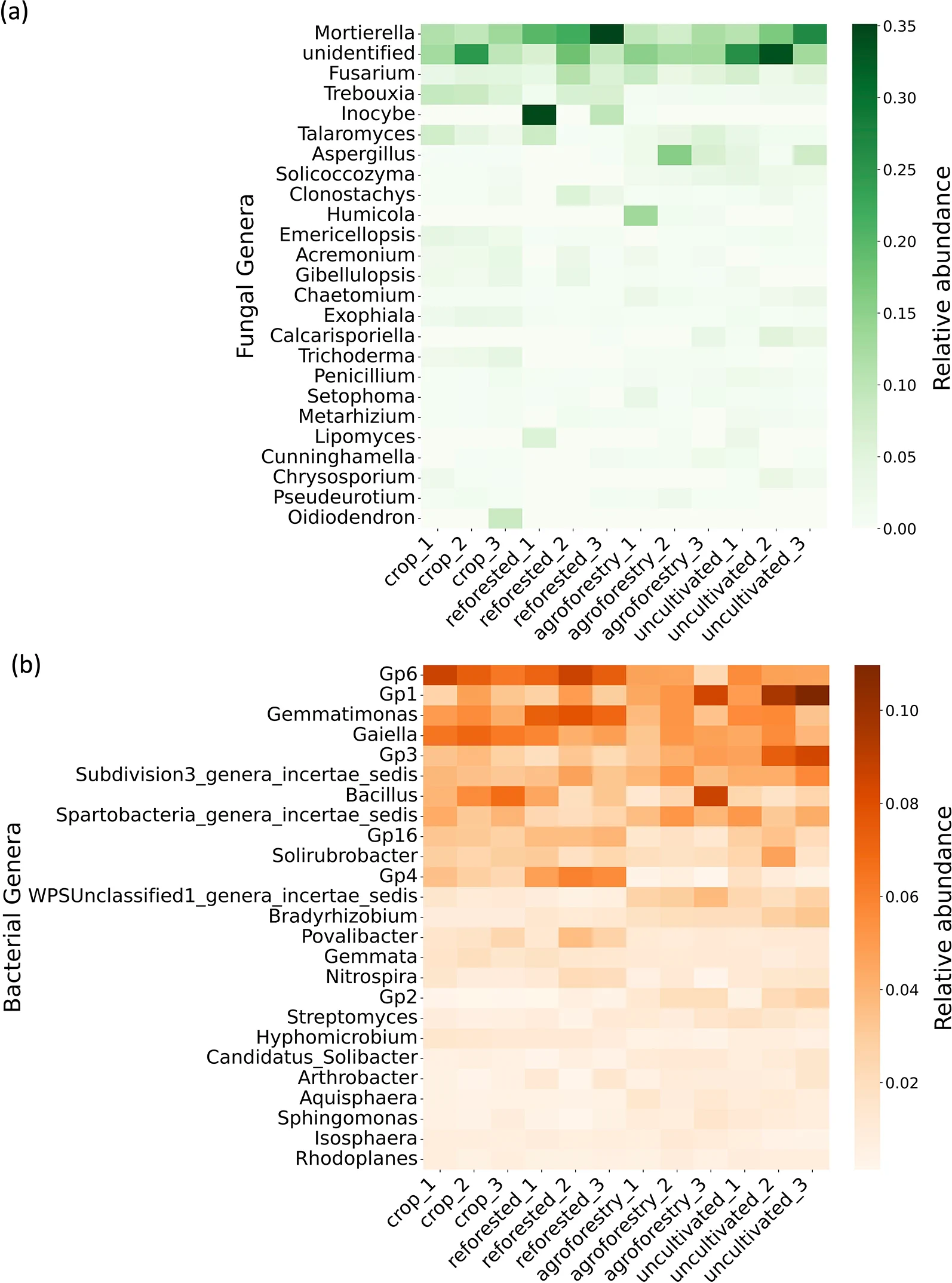
Heatmaps of (a) the 25 most abundant fungal genera, and of (b) the 25 most abundant bacterial genera across 12 soil samples representing four land-use types (crop, reforested, agroforestry, and uncultivated). Genera are ordered by overall abundance. Color intensity reflects relative abundance within each sample.

To characterise differences in soil communities across land-use types, we analysed the taxonomic composition, richness, and diversity of fungal and bacterial communities, and quantified the overlap in species occurrence among land uses. Heatmap visualisation of the 25 most abundant fungal and bacterial genera revealed clear differences in taxonomic profiles among the four land-use types ( Figure 1). Fungal communities showed stronger variation in dominant genera across land uses than bacterial communities, with certain genera significantly more abundant in specific samples and in specific land-use types; for instance, Mortierella and Inocybe are much more abundant in reforested than in any other soils. In contrast, the relative abundance in bacterial profiles displayed a higher homogeneity among samples, although clustering within the same land-use type is still visible to a certain degree. We note that Gp6, Gemmatimonas, and Gaiella are more abundant in crop and reforested soils, while Gp1 and Gp3 are more present in uncultivated soils. In general, fungal samples were dominated by genera such as Mortierella, Fusarium, and Trebouxia, while bacterial communities showed high relative abundances of members of the Acidobacteria group, Gemmatimonas and Gaiella. It is worth noting that in both fungal and bacterial datasets, some of the most abundant features were not fully resolved to genus level, suggesting the presence of a substantial fraction of community members that remain taxonomically undercharacterised. These results underline the stronger sensitivity of fungal communities to land-use and land-use change, probably due to their symbiotic associations with plants and strong linkage with organic matter, namely lignin and complex organic molecules, in undisturbed conditions. ^17^ From an agroecological perspective, the observed land-use–associated differences in fungal diversity are consistent with the idea that management practices influencing soil organic matter and nutrient dynamics may contribute to shaping fungal communities.

**Figure 2.**
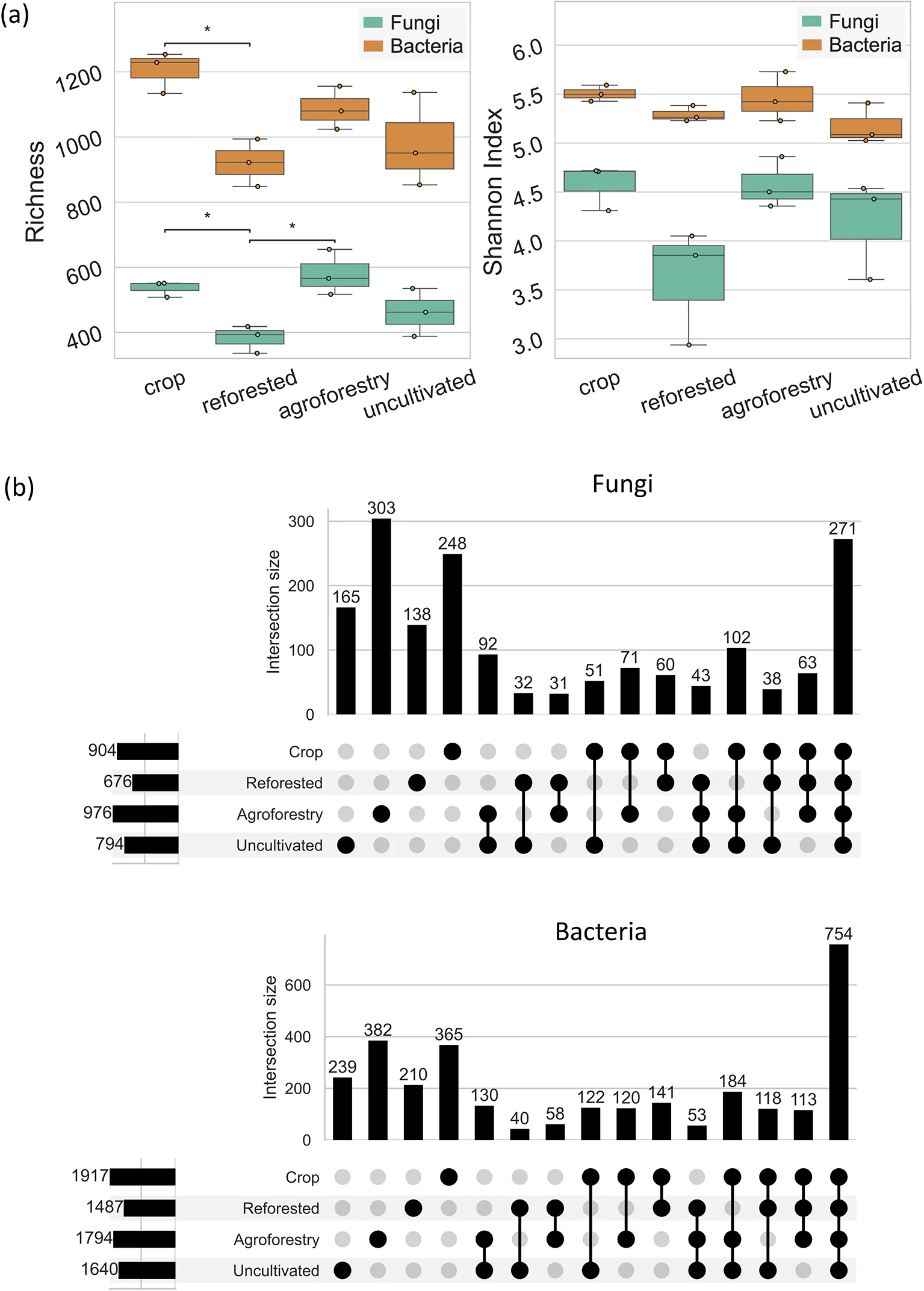
(a) Boxplots of fungal and bacterial richness and Shannon’s diversity index at species level across different land uses. Significant differences between land-uses, assessed with a Tukey HSD test on the mean values of each ecological index, are indicated with a bar over the couple of boxplots and a number of stars. Differences are considered statistically significant at p-value *<*0.05 and are reported with the following legend: 10 ^−2^ *< p <* 5 *·* 10 ^−2^ (*), 10 ^−3^ *< p <* 10 ^−2^ (**), 10 ^−4^ *< p <* 10 ^−3^ (***), *p <* 10 ^−4^ (****). (b) UpSet plots showing the intersection of bacterial and fungal species across four land use types. Each plot illustrates the number of species unique to or shared between different land uses. Horizontal bars represent the total number of species observed per land use, while the vertical bars show the size of species sets defined by each intersection.

**Figure 3.**
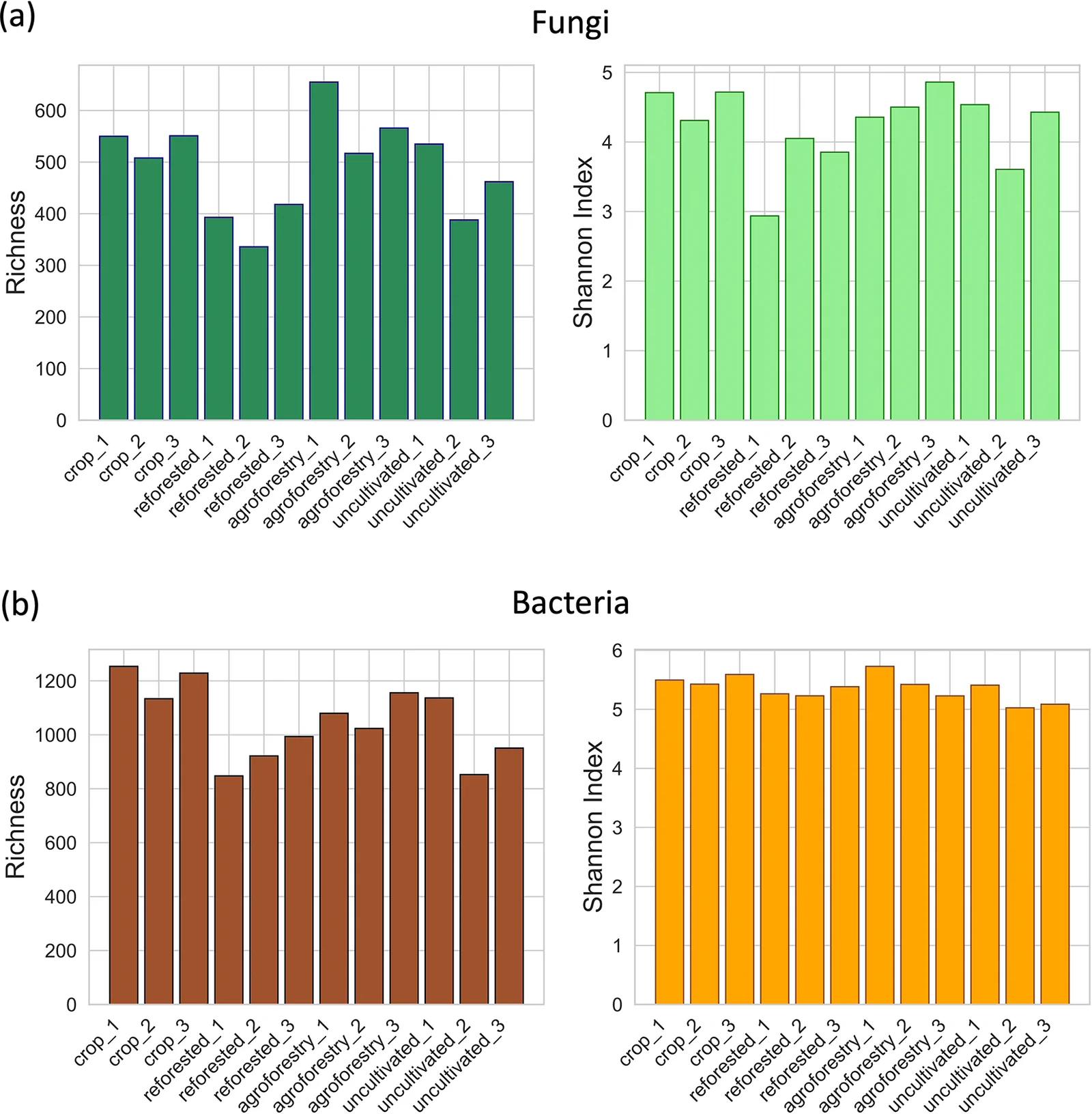
Ecological indices of fungal and bacterial communities across land-uses. ( **a**) Richness (left) and Shannon diversity index (right) for fungal communities in 12 soil samples. ( **b**) Richness (left) and Shannon diversity index (right) for bacterial communities in the same samples. Each bar represents a replicate within one of four land-use categories: crop, reforested, agroforestry, and uncultivated.

Comparisons of diversity metrics at the species level showed that both fungal and bacterial richness and Shannon diversity varied noticeably across land-use types ( Figure 2a). Uncultivated and reforested soils consistently exhibited lower richness than crop and agroforestry. Formal statistical testing (n = 3 per land-use type) detected significant differences only for richness: for bacteria, between crop and reforested soils (p = 2.3 × 10 ^−2^) and for fungi, between crop and reforested soils (p = 4.0 × 10 ^−2^) as well as between reforested and agroforestry soils (p = 1.1 × 10 ^−2^). No significant differences were detected for Shannon diversity among land-use types. Barplots showing patterns of richness and Shannon diversity across replicates are displayed in Figure 3, which confirms that the trends observed in the boxplots are consistent at the sample level.

**Figure 4.**
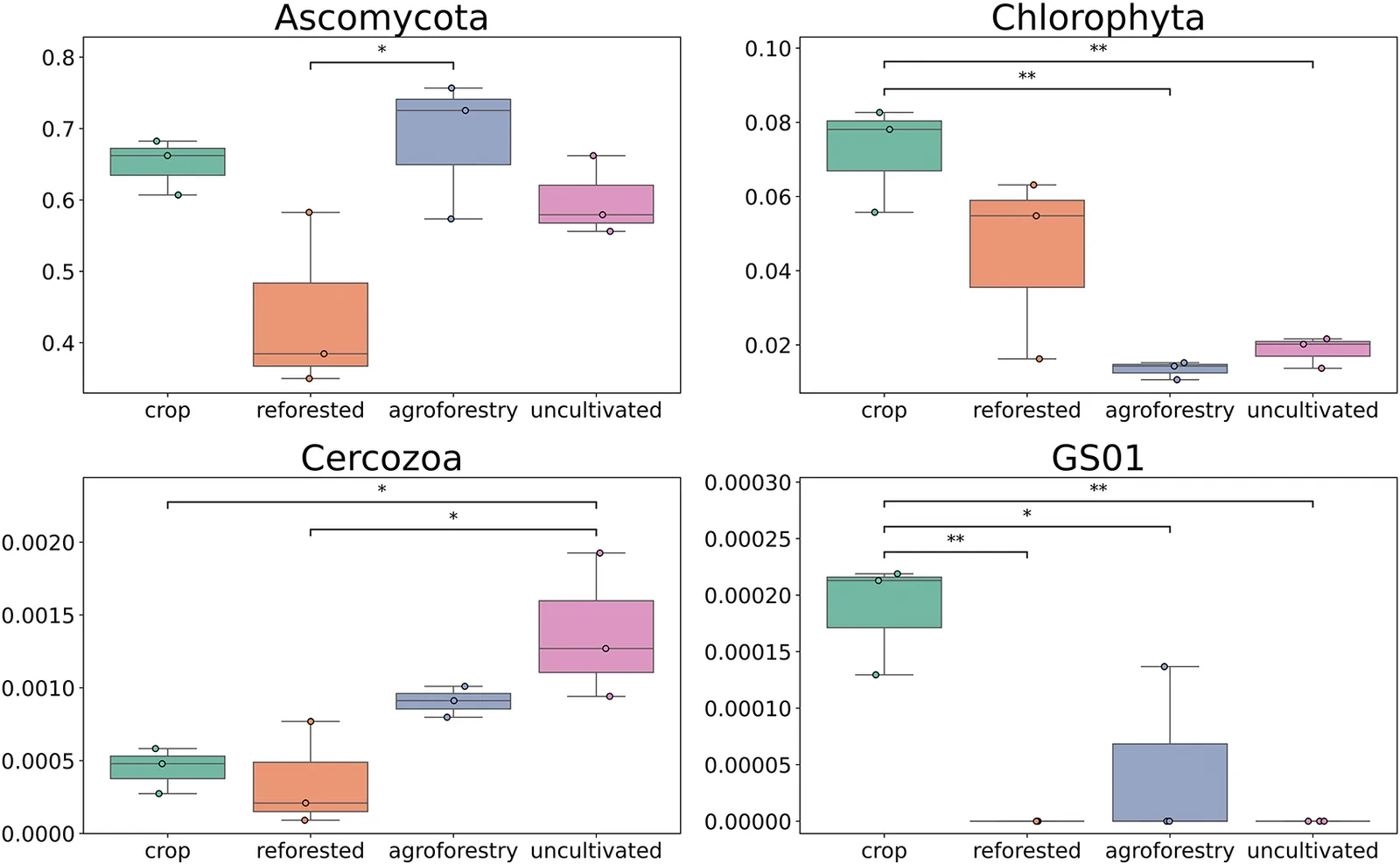
Boxplots of relative abundance of selected fungal genera across land-use types. Only taxa with statistically significant differences (assessed with Tukey HSD test) are shown. Differences are considered statistically significant at p-value *<*0.05 and are reported with the following legend: 10 ^−2^ *< p <* 5 *·* 10 ^−2^ (*), 10 ^−3^ *< p <* 10 ^−2^ (**), 10 ^−4^ *< p <* 10 ^−3^ (***), *p <* 10 ^−4^ (****).

**Figure 5.**
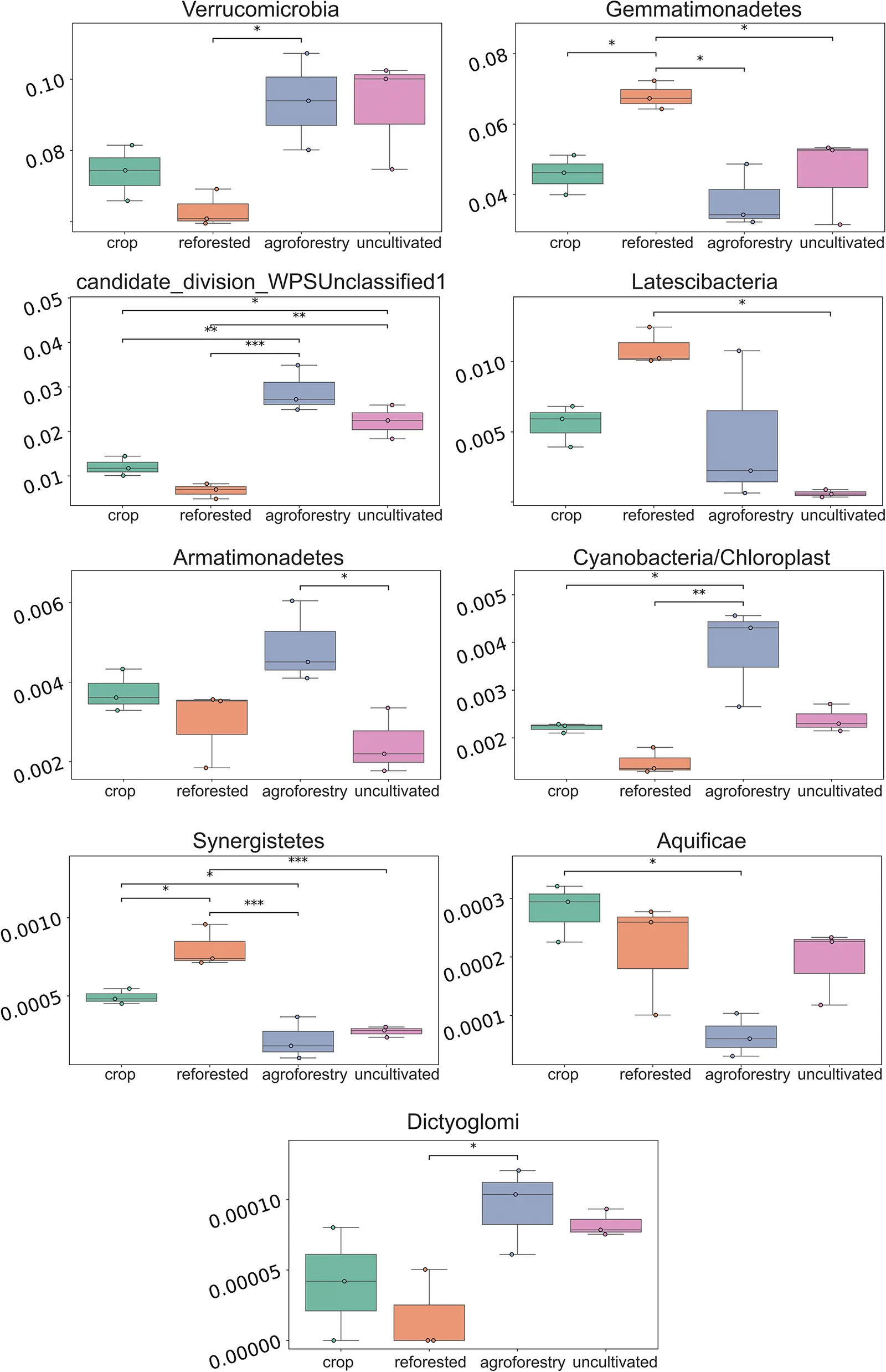
Boxplots of relative abundance of selected bacterial genera across land-use types. Only taxa with statistically significant differences (assessed with Tukey HSD test) are shown. Differences are considered statistically significant at p-value *<*0.05 and are reported with the following legend: 10 ^−2^ *< p <* 5 *·* 10 ^−2^ (*), 10 ^−3^ *< p <* 10 ^−2^ (**), 10 ^−4^ *< p <* 10 ^−3^ (***), *p <* 10 ^−4^ (****).

UpSet plots of species presence–absence ( Figure 2b) showed that each land-use type contained unique fungal and bacterial taxa, but agroforestry soils had the highest number of exclusive taxa in both kingdoms, whereas reforested soils had the fewest. This suggests that agroforestry management is associated with increased compositional variety and may increase soil biodiversity relative to the other land uses examined. Significant overlap in community composition was observed, especially in bacteria, with over 700 species common to all the land-use types. In addition to species-level differences, significant shifts were also observed at higher taxonomic ranks. To evaluate compositional diversity across land-uses, we then computed Whittaker beta diversity within and between land-uses for fungal and bacterial communities. Within each land use, β(W) = γ/α̅ was consistently >1 for both kingdoms, indicating moderate taxonomic turnover among the three biological replicates. Turnover among replicates was generally higher for fungi than for bacteria. Pairwise comparisons between pooled land-use communities revealed moderate turnover across all land-use pairs, with the highest dissimilarity involving agroforestry and reforested soils. Together, these patterns suggest that land-use type is associated with compositional differences in both fungal and bacterial communities. Figure 4 and Figure 5 show fungal and bacterial phyla whose relative abundances varied significantly across land-use types, indicating that land-use effects are strong also at broader taxonomic groups. Notably, a larger number of bacterial phyla showed significant differences compared to fungi, likely reflecting the higher overall richness and taxonomic diversity of bacterial communities.

### Correlation patterns among samples and abiotic parameters

**Figure 6.**
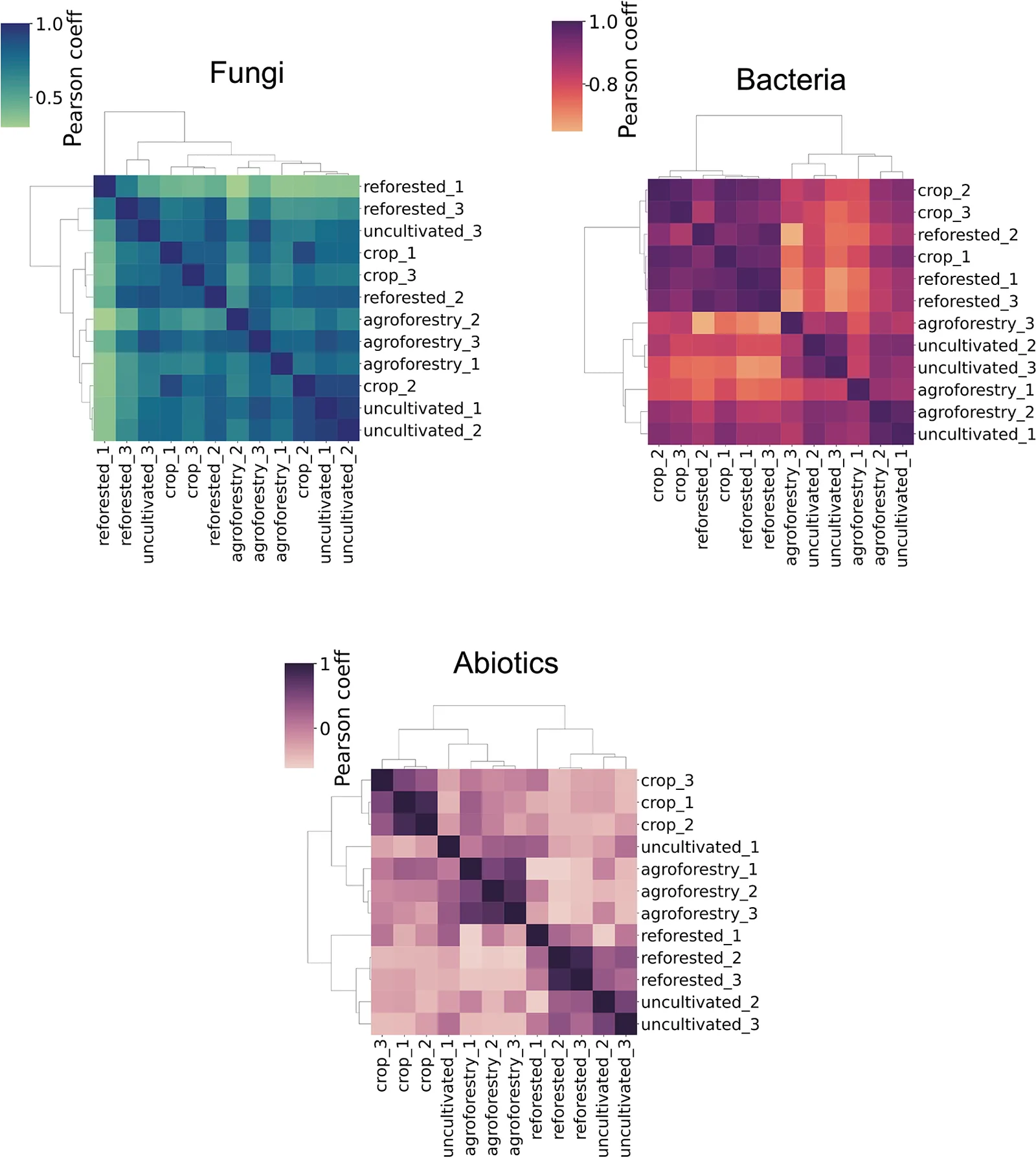
Heatmaps of pairwise Pearson correlations among samples based on fungal, bacterial, and abiotic profiles. Each panel displays hierarchical clustering of the 12 soil samples based on their correlations. Darker colors indicate stronger positive correlations.

To explore similarities among samples, we computed Pearson correlation matrices based on the bacterial, fungal and abiotic profiles of each sample; correlation matrices were represented as clustered heatmaps ( Figure 6). In all three cases, samples sharing the same land-use tend to cluster; bacterial communities were more strongly clustered by land use than fungal communities, with two clear clusters formed by crop-reforested and uncultivated-agroforestry samples.

**Figure 7.**
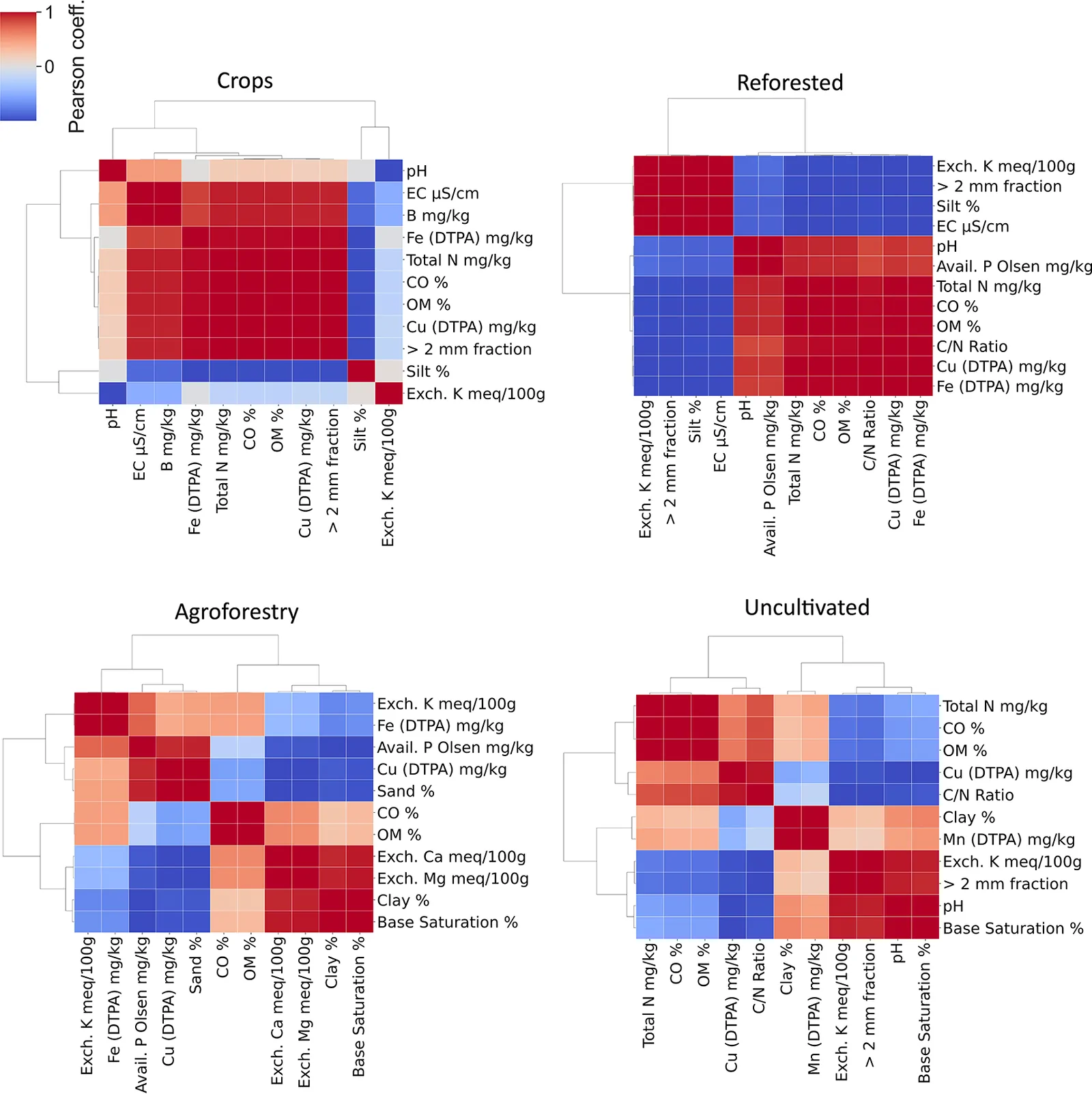
The figure displays a clustered heatmap of the top 20 strongest pairwise Pearson correlations (by absolute value) among abiotic variables. Only variables involved in at least one of these top correlations were retained in the plot. The color scale represents the Pearson correlation coefficient.

Moreover, we computed the Pearson correlation matrices of the abiotic parameters to investigate relationships among chemical and physical soil properties. Analysis of the top 20 strongest pairwise correlations among abiotic variables ( Figure 7) revealed different patterns across land-use types: crop and reforested soils showed more compact correlation clusters dominated by strong co-variation among fertility-related variables (e.g., organic matter, total nitrogen, and organic carbon), whereas agroforestry and uncultivated soils displayed more heterogeneous patterns with multiple smaller clusters. In these two land uses, several subgroups of variables emerged, leading to a higher structural complexity. The complete correlation matrices (showing all the correlations, not only the strongest ones) for the different land-use types are shown in Figure 9– Figure 12. These plots display patterns broadly consistent with those observed in the reduced correlation matrices.

We also assessed significant differences for each abiotic parameter across land-use types; the parameters showing the strongest effects are reported in Figure 13.

### Multivariate analysis of soil communities and abiotic parameters

**Figure 8.**
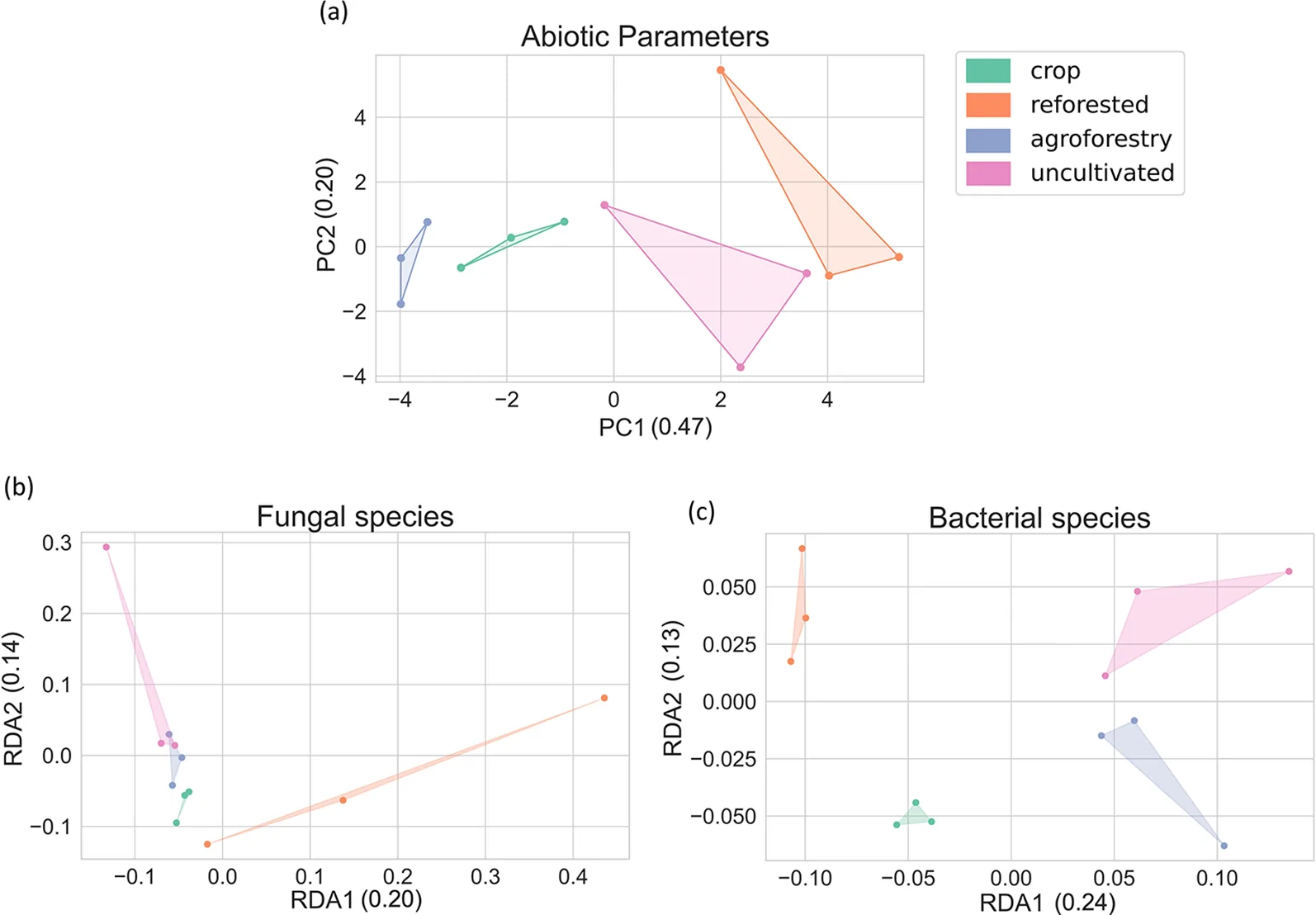
Ordination plots of abiotic and species data. ( **a**) Principal Component Analysis (PCA) of abiotic parameters across samples. ( **b**) Redundancy Analysis (RDA) of fungal species constrained by selected abiotic variables. ( **c**) Redundancy Analysis (RDA) of bacterial species constrained by selected abiotic variables. Convex hulls connect replicates within each land use group to visually highlight the clusters. The values between parentheses report the variance explained by each component.

In order to assess how abiotic parameters shape microbial community structure, we performed ordination analyses. Principal Component Analysis (PCA) of abiotic variables ( Figure 8a) explained a substantial fraction of variance in the first two components ( *>* 60%) and showed clear separation of samples according to land use. Top abiotic contributors to the first principal component were Cation-Exchange Capacity, Total Nitrogen and Boron. These soil features are closely linked to the management of organic matter and organic carbon, indicating that land-use differences align with a gradient in nutrient status and related soil properties. Agronomic practices aimed at increasing organic inputs and enhancing specific nutrient cycles are crucial for positively shaping soil microbiomes.

**Figure 9.**
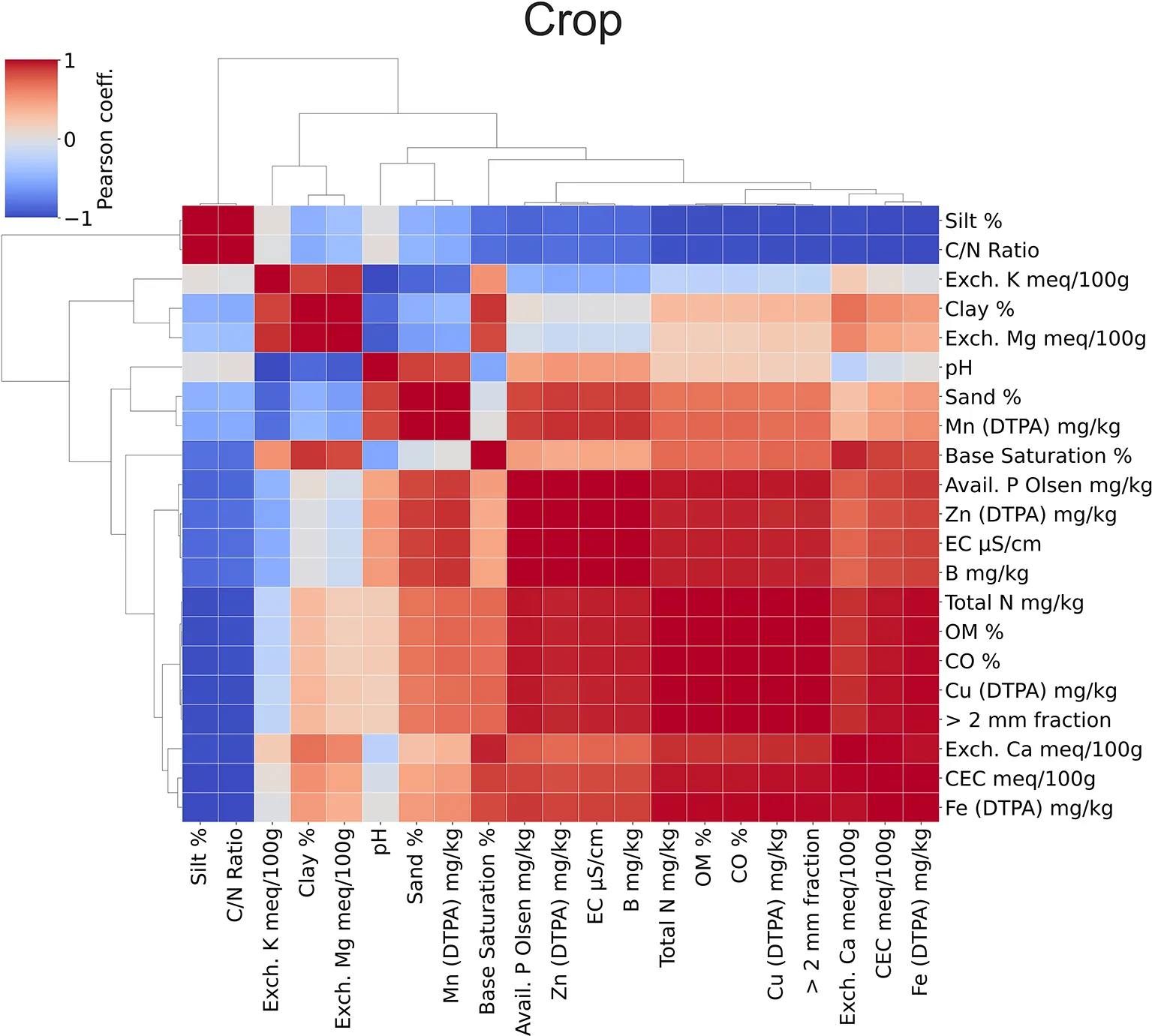
Heatmaps of pairwise Pearson correlations among abiotic parameters in Crop land-use. Each panel displays hierarchical clustering of the abiotic parameters based on their correlations (for abbreviation key see Table 2). Darker colors indicate stronger positive correlations.

**Figure 10.**
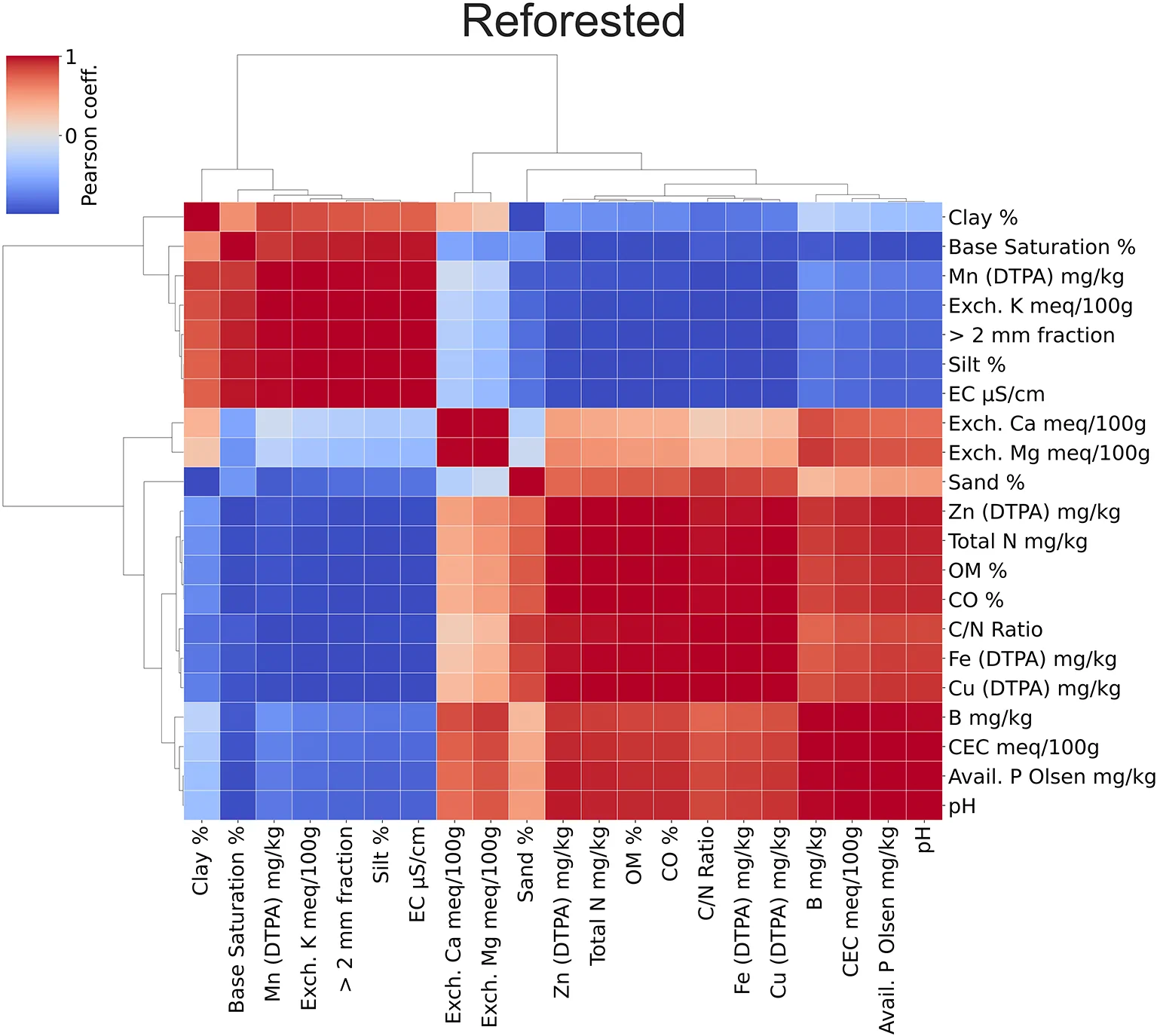
Heatmaps of pairwise Pearson correlations among abiotic parameters in Reforested land-use. Each panel displays hierarchical clustering of the abiotic parameters based on their correlations (for abbreviation key see Table 2). Darker colors indicate stronger positive correlations.

**Figure 11.**
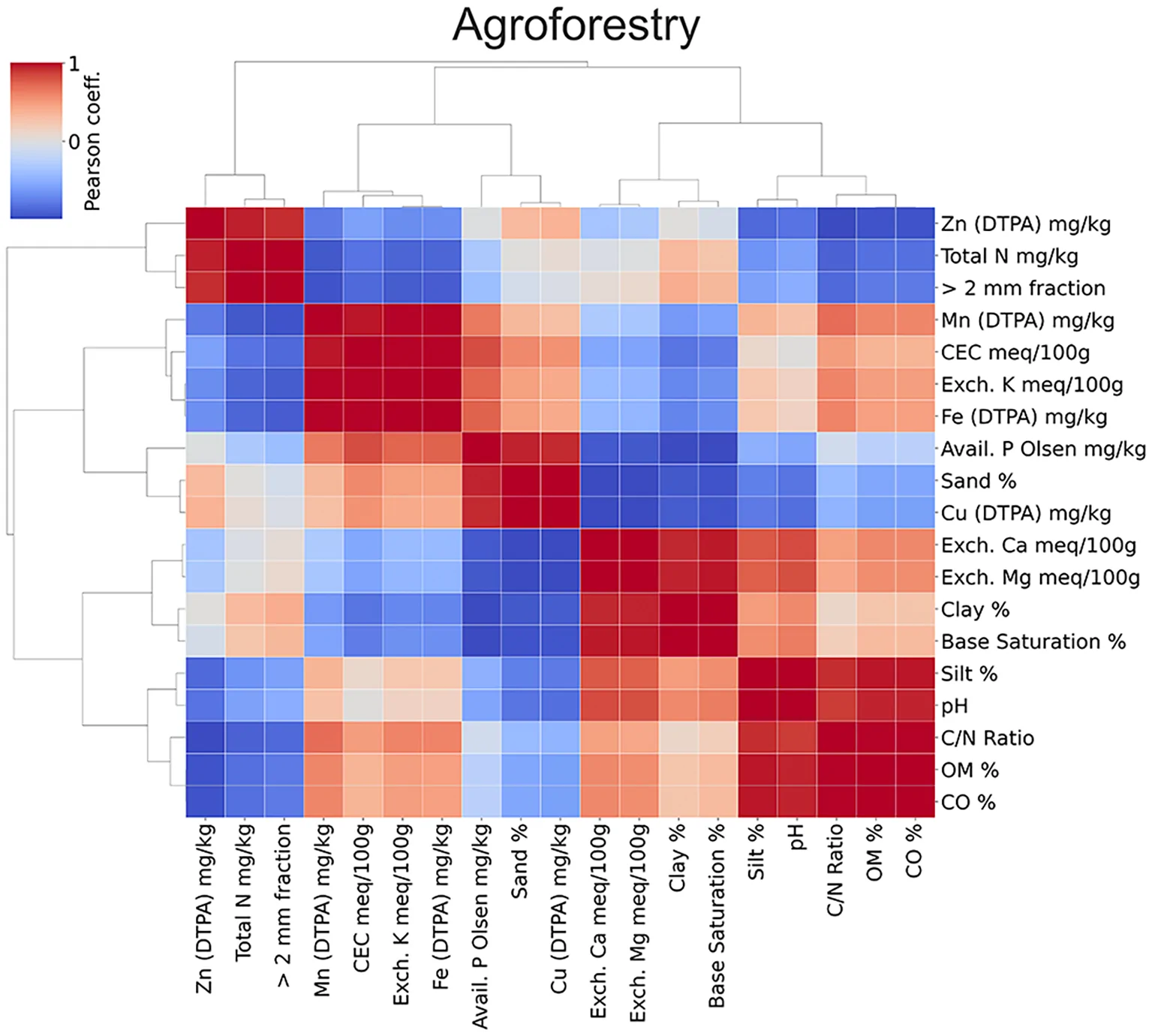
Heatmaps of pairwise Pearson correlations among abiotic parameters in Agroforestry land-use. Each panel displays hierarchical clustering of the abiotic parameters based on their correlations (for abbreviation key see Table 2). Darker colors indicate stronger positive correlations.

**Figure 12.**
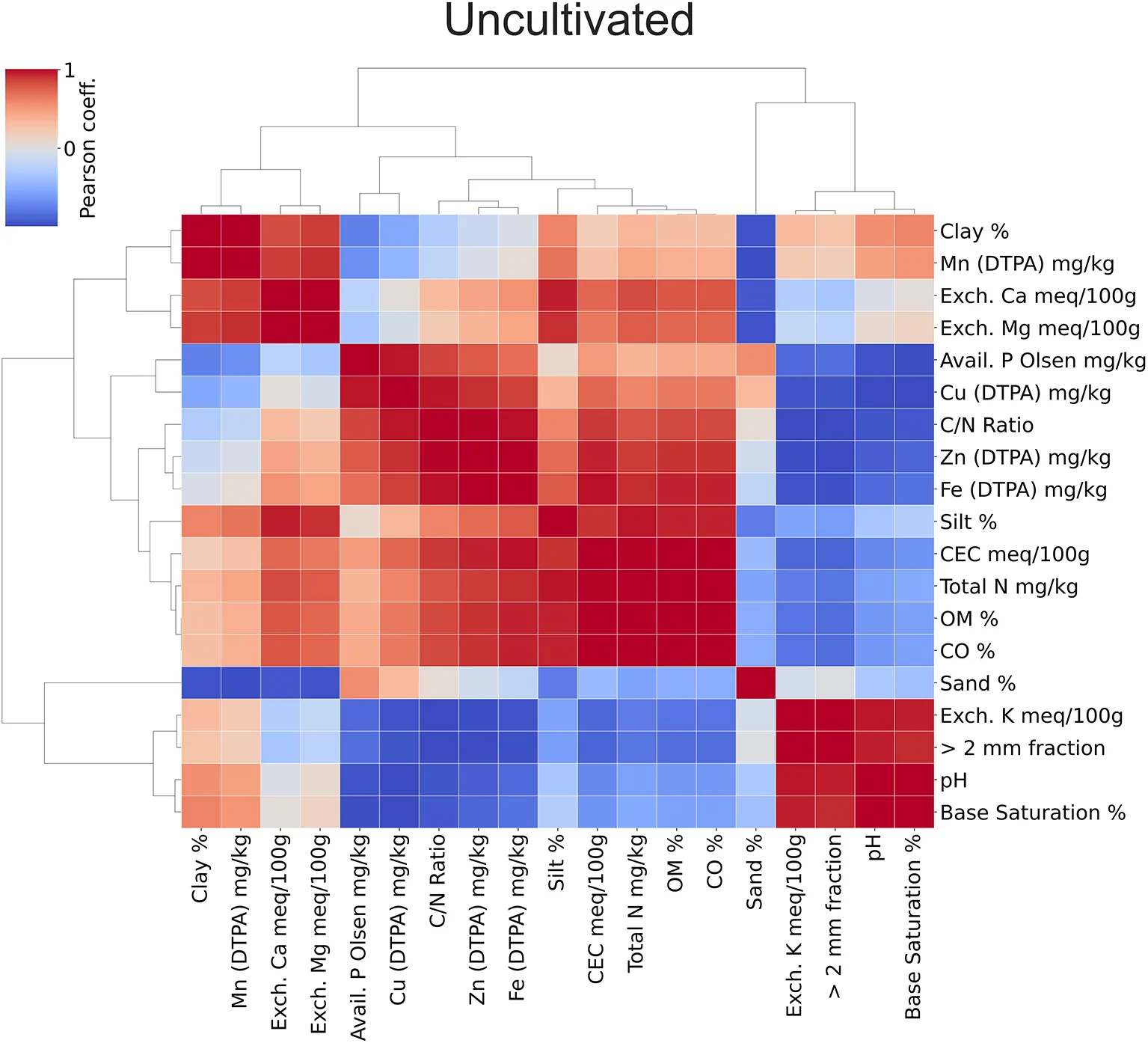
Heatmaps of pairwise Pearson correlations among abiotic parameters in Uncultivated land-use. Each panel displays hierarchical clustering of the abiotic parameters based on their correlations (for abbreviation key see Table 2). Darker colors indicate stronger positive correlations.

**Figure 13.**
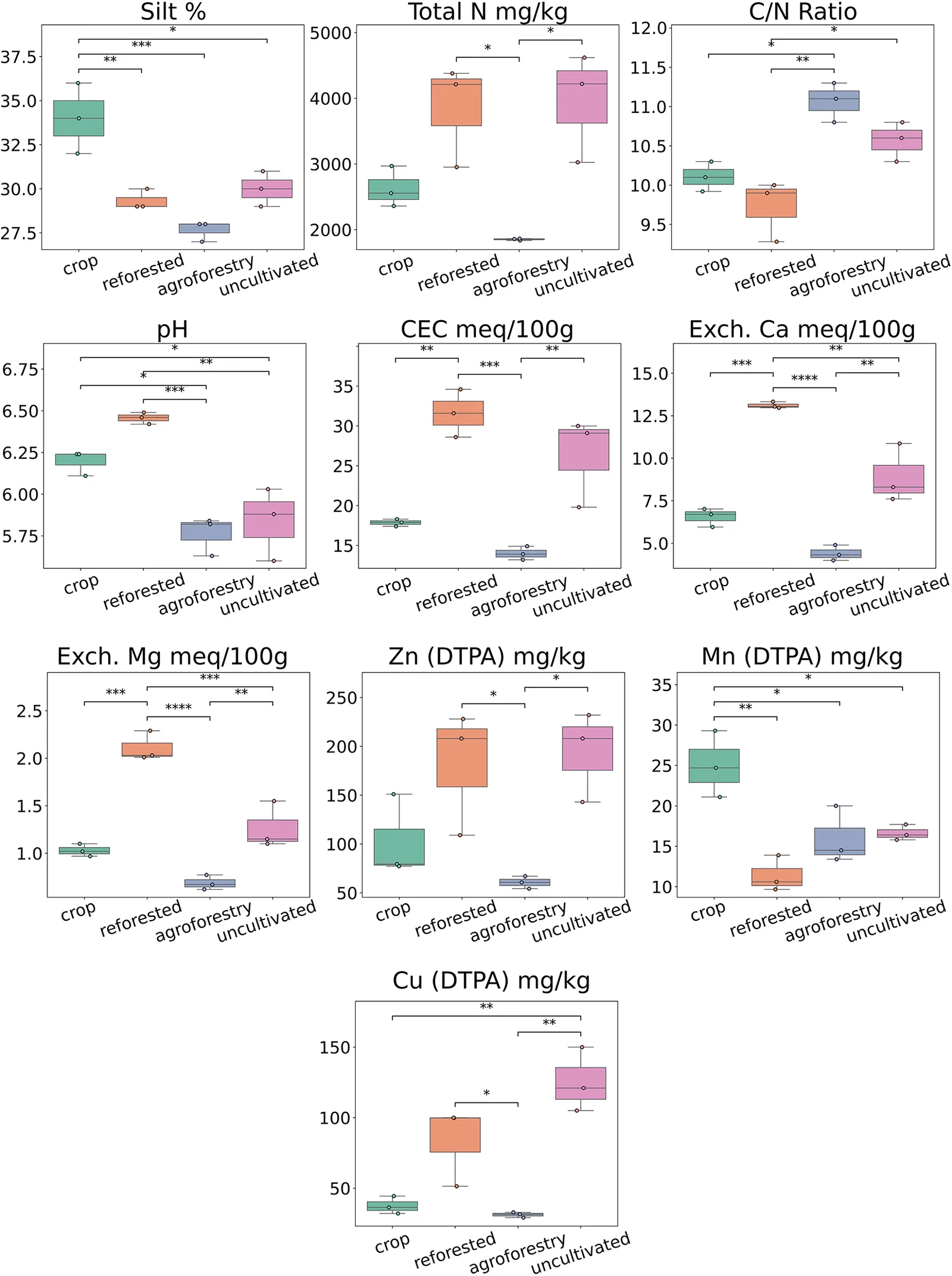
Boxplots of abiotic parameters across land-use types. Only parameters with statistically significant differences (assessed with Tukey HSD test) are shown. Differences are considered statistically significant at p-value *<*0.05 and are reported with the following legend: 10 ^−2^ *< p <* 5 *·* 10 ^−2^ (*), 10 ^−3^ *< p <* 10 ^−2^ (**), 10 ^−4^ *< p <* 10 ^−3^ (***), *p <* 10 ^−4^ (****). Abbreviation keys are reported in Table 2).

**Figure 14.**
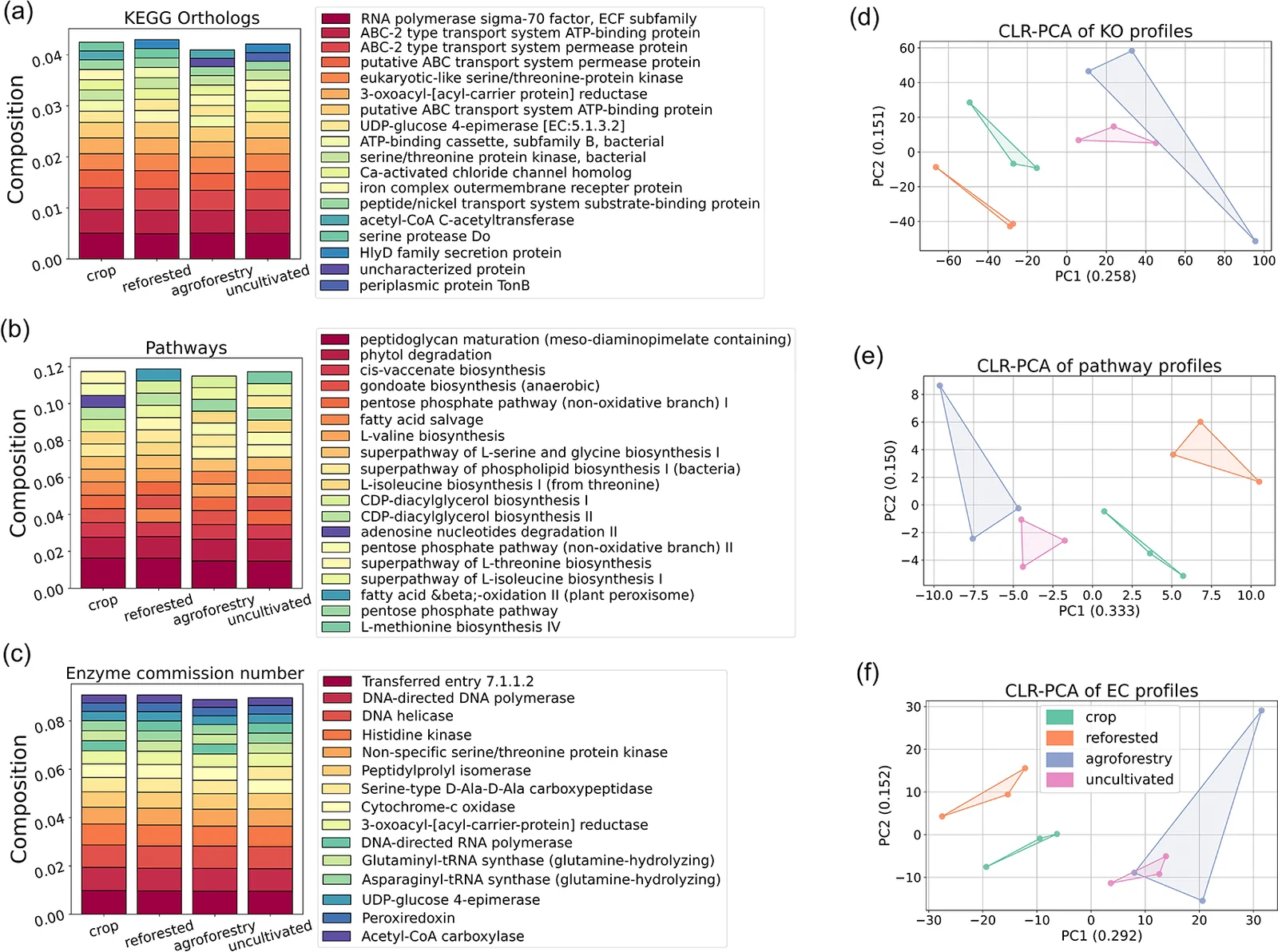
(a)(b)(c) Stacked plots of the 15 most represented functions as expressed by KEGG, EC and MetaCyc metabolic pathways across land-use type. (d)(e)(f) Principal Component Analysis (PCA) of bacterial KEGG, EC and MetaCyc metabolic pathways functional profiles across land-use type, as obtained from PICRUSt2 analysis. Convex hulls connect replicates within each land use group to visually highlight the clusters. The values between parentheses report the variance explained by each component.

**Figure 15.**
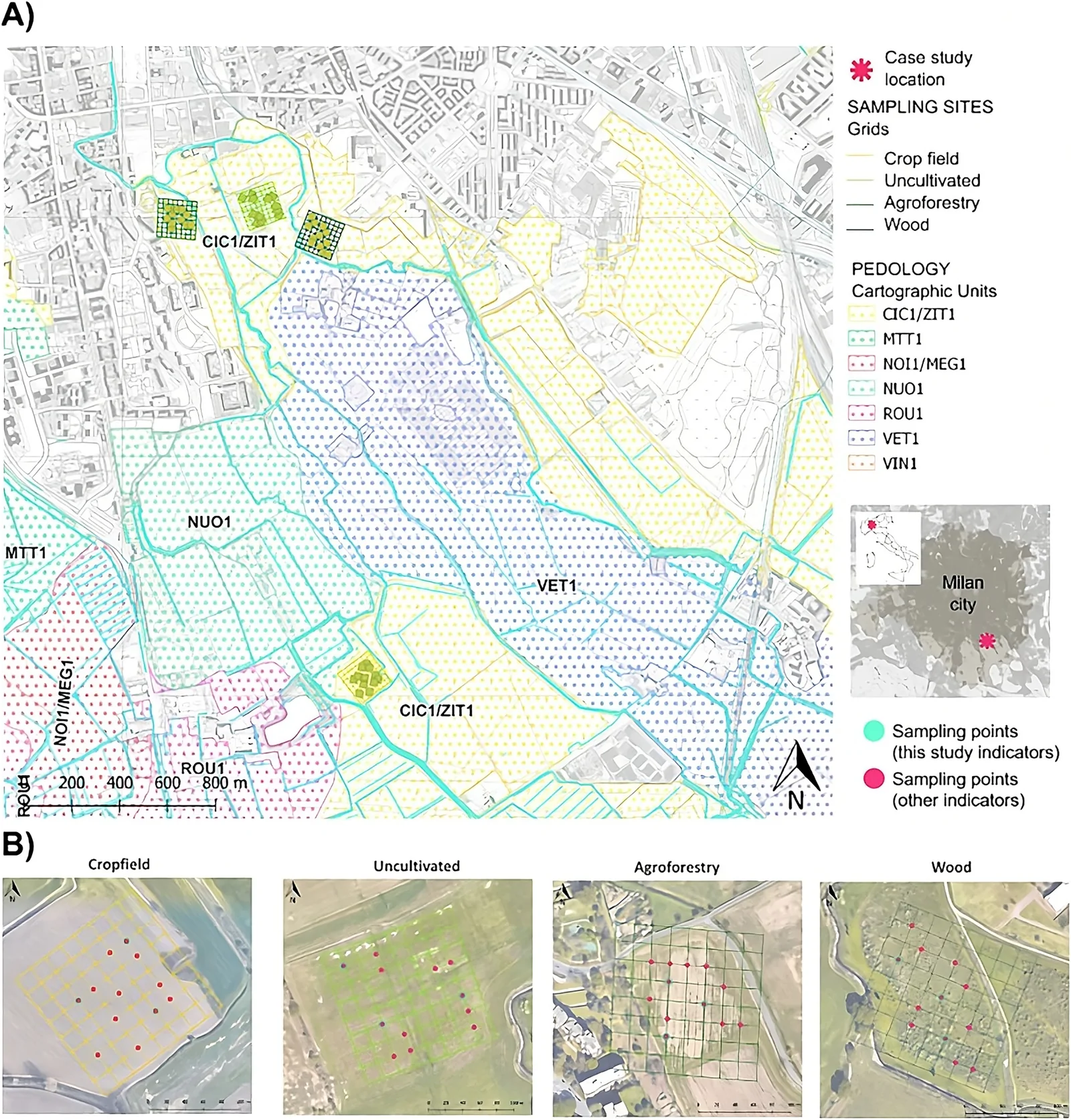
a) The study area location in Milan metropolitan area and sampling sites location in CIC1/ZIT1 Soil Cartographic Unit - Cambisols; b) sampling sites location and sampling protocol.

Redundancy Analysis (RDA) of fungal and bacterial taxonomical profiles, where abiotic parameters were used as explanatory variables, confirmed significant associations between abiotic factors and soil community structure. The first 2 components of the RDA explained more than 30% of the variance, and both fungal and bacterial species were found to cluster in this 2-dimensional space, although this result is much more evident for bacteria. Vectors of the explanatory variables (CEC, B, exchangeable Ca and Mg, and OM) define the main constrained gradient. In the fungal RDA, the reforested samples project in the same direction as the vectors for CEC and exchangeable base cations (Ca, Mg), indicating that fungal community differences among land uses are associated with these fertility-related parameters. This interpretation is consistent with the abiotic boxplots, where reforested soils show higher CEC, exchangeable Ca, and exchangeable Mg, compared with the other land uses. In the bacterial RDA, the most prominent feature is the alignment of agroforestry points with exchangeable Ca and Mg vectors.

### Functional analysis of bacterial communities

To investigate whether land-use type influences microbial functional potential, we used PICRUSt2 to predict bacterial functional profiles; in particular, we computed Kyoto Encyclopedia of Genes and Genomes (KEGG) Orthology (KO) terms, Enzyme Commission (EC) numbers, and MetaCyc metabolic pathways for each land-use. Principal component analysis of these profiles consistently separated the different land-uses ( Figure 14a–c), although uncultivated and agroforestry EC profiles partially overlap in the 2-dimensional space defined by the first 2 components of the PCA. We also created stacked bar plots of the 15 most abundant functions ( Figure 14d–f) for each functional profile across the different land-uses. These stacked plots represent about 10% of the total predicted functional profile, indicating that the vast majority of detected functions occur at lower relative abundances. Moreover, the most prevalent functions displayed broadly similar relative abundances across land-use types, suggesting that a common set of core metabolic capacities dominates bacterial communities regardless of management regime; land-use–associated differences emerge primarily in the multivariate structure of the predicted functional profiles and in the long tail of lower-abundance functions. In particular, at the pathway level, reforested soils showed a comparatively higher contribution of fatty-acid β-oxidation II (plant peroxisome), whereas uncultivated soils displayed a slightly greater representation of pathways related to central metabolism and biosynthesis (e.g., pentose phosphate pathway and L-methionine biosynthesis IV). In contrast, cropland samples tended to show marginally higher contributions for adenosine nucleotides degradation II.

## Methods

### Study area

The study is applied to an agricultural area on the southeastern edge of the city of Milan ( Figure 1), which has been the focus of recent strategic transformation plans aimed at establishing an agroecological experimentation laboratory, ^18^ integrated into the Living Lab Milano Porta Verde system: ^19^ an open-air laboratory dedicated to the socio-environmental regeneration of the area. The study area covers approximately 100 ha, part of the South Milan Agricultural Park - Vettabbia Valley system, an ancient strategic axis linking city and countryside. ^20^ The area is predominantly composed of agricultural lands (arable crops, horticulture, permanent and fallow grasslands) and semi-natural areas (young reforestation). Since 2019, the implementation of 2 ha under productive agroforestry management - a multilayered agro-silvo-pastoral system inspired by regenerative practices - has begun ( Figure 1). The study area belongs to an alluvial context (Po Plain) dominated by Cambisols (WRB Classification System ^21^), and Gleysols strips along water courses. A loamy-sandy texture predominates: loose and relatively permeable soils, easily workable, typical of alluvial contexts. Soil samples were collected 16–17 April 2023 for DNA analysis while samples for soil abiotic analysis were collected 27 June 2023. Four land use types were investigated as treatments: crop field (crop), uncultivated area (uncultivated), wooded area (reforested), agroforestry system (agroforestry) (a summary of management history (current management, previous land use, products) is included in Table 1). Their selection was based on:

• the intention to compare the agroforestry system with different local land uses, yet still of agro-silvo-pastoral or semi-natural type;
• the opportunity to compare sites characterized by the same pedological substrate (same cartographic Unit ^22^).

**Table 1.** Sampling sites main features.

Land use type	Abbr.	Current management	Current management start date	Previous land use	Products
Annual crop field	Crop	Conservation agriculture	About 25 years ago	Conventional crop field	Corn (2–3 yrs) in rotation with winter wheat (1 yr) and soyabean (1 yr)
Wooded area	Reforested	Semi-natural	2018 (planting)	Uncultivated area	No products
Agroforestry system	Agroforestry	Regenerative agriculture	2019 (planting)	Conventional crop field	Fruits, berries, eggs
Uncultivated area	Uncultivated	Mowings	Stable for decades	Crop field	Hay

In each site, sampling was conducted at selected nodes of an 8 × 8 grid with a 20 m mesh ( Figure 15): we sampled randomly in each grid, for a total of 3 soil sampling points for each site, avoiding marginal points, which often coincided with the edges of the site (roads, ditches, tree rows) and were more likely to be subject to disturbances that could influence the analytical results. Points that coincided with specific sources of heterogeneity within the site were also excluded, such as those located very close ( *<* 2 m) to tree rows crossing the uncultivated area. The sampling was carried out by removing litter and picking soil slices through a spade at a depth of 0–30 cm with fixed thickness. Table 2 reports the soil parameters investigated. Abiotic parameters were measured at Labs & Technological Services AGQ, S.L. ( https://agqlabs.com/en/).

**Table 2.** Abiotic parameters: complete names and abbreviations used in figures.

Abiotic parameter	Abbreviation
Sand %	Sand %
Clay %	Clay %
Silt %	Silt %
Total Nitrogen mg/kg	Total N mg/kg
C/N Ratio	C/N Ratio
Organic Matter %	OM %
OC %	OC %
Available Phosphorus Olsen mg/kg	Avail. P Olsen mk/kg
pH	pH
Electrical Conductivity ( *μ*S cm ^−1^, 20 °C)	EC *μ*S/cm
Cation-Exchange Capacity meq 100 g ^−1^	CEC meq 100 g ^−1^
Base Saturation %	Base Saturation %
Exchangeable Calcium meq/100 g	Exch. Ca meq/100 g
Exchangeable Potassium meq/100 g	Exch. K meq/100 g
Exchangeable Magnesium meq/100 g	Exch. Mg meq/100 g
Zinc (DTPA) mg/kg	Zn (DTPA) mg/kg
Manganese (DPTA) mg/kg	Mn (DTPA) mg/kg
Iron (DTPA) mg/kg	Fe (DTPA) mg/kg
Boron (mg/kg)	B mg/kg
Copper (DTPA) mg/kg	Cu (DTPA) mg/kg
> 2 mm fraction	> 2 mm fraction

### Metagenomic sequencing library preparation, sequencing and analysis

The next generation sequencing experiments, which included quality control and the initial bioinformatics analysis, were executed by Genomix4life S.R.L. (Baronissi, Salerno, Italy). DNA quality and quantity were checked with the NanoDropOne spectrophotometer (Thermo Scientific, Waltham, MA, USA) and the Qubit Fluorometer 4.0 (Invitrogen Co., Carlsbad, CA, USA). 16S amplification was performed using primers targeting the hypervariable V3 and V4 region of the 16S rRNA gene 314F(5’-CCTACGGGNGGCWGCAG-3′) and 806R (5’-GACTACHVGGGTATCTAATCC-3′), ^23^ while ITS amplification used the ITS3 – ITS4 primers (ITS3f 5′- GCATCGATGAAGAACGCAGC -3’ ITS4r: 5’-TCCTCCGCTTATTGATATGC -3′). PCR amplification and library preparation followed the Illumina 16S Metagenomic Sequencing Library Preparation protocol (Illumina, San Diego, CA, USA). Briefly, a two-step PCR protocol was used: the first PCR amplified the V3–V4 region of the bacterial 16S rRNA gene using locus-specific primers containing Illumina overhang adapters, while the second PCR attached dual indices and sequencing adapters using the Nextera XT Index Kit (Illumina). Amplifications were performed in duplicate for each sample to reduce PCR bias and the resulting products were pooled prior to library preparation. A negative control, including all reagents used in amplification and library preparation but excluding DNA template, was included to monitor potential contamination. The negative control included during library preparation did not generate a detectable library profile. During the sequencing run no reads were obtained from the negative control sample. Therefore, no sequences from the negative control were present or required filtering in the downstream analyses. Libraries were quantified using a Qubit fluorometer (Invitrogen Co., Carlsbad, CA, USA) and pooled to obtain an equimolar concentration of 4 nM for each index-tagged sample, with the addition of the PhiX Control Library. The pooled libraries underwent cluster generation and sequencing on the MiSeq platform (Illumina) using a 2X250 bp paired-end format. The amplicon sequencing data were analyzed using the 16S Metagenomics application available in the Illumina BaseSpace Sequence Hub. This pipeline performs read processing and taxonomic classification using a k-mer–based Naïve Bayesian classifier derived from the Ribosomal Database Project (RDP) algorithm. The RefSeq RDP 16S v3 database was used for taxonomic assignment of 16S rRNA sequences. For the ITS datasets, the same analytical framework was used; however, taxonomic assignment was performed using the UNITE reference database, which is specifically curated for fungal ITS sequences. Therefore, the pipeline itself was not fundamentally different, but the reference database used for taxonomic classification was adapted to account for the differences between bacterial 16S and fungal ITS markers. The UNITE Fungal ITS Database v7.2 is based on FASTA from https://doi.org/10.15156/BIO/587475 Includes singletons set as RefS (in dynamic files).

### Community diversity and taxonomical composition

Raw taxa abundances were first processed by normalizing the values for each sample to relative abundances (ranging from 0 to 1). To visualise the composition of the most common taxa at genus-level, we generated heatmaps showing the relative abundance of the 25 most abundant fungal and bacterial genera across the 12 soil samples.

To quantitatively assess the composition of soil communities, we calculated two alpha-diversity metrics: observed richness (number of unique species) and Shannon diversity index.

These metrics were computed separately for fungal and bacterial taxa, based on species-level metagenomic assignments from each of the 12 soil samples.

Richness was defined as the count of distinct taxa detected per sample. The Shannon diversity index was calculated as:

Hi=−Lpii=1Nlnpi, (1)

where *N* is the number of observed taxa in a sample and *p _i_* is the relative abundance of the *i*th taxon. To visualise the ecological indices, we created barplots of richness and Shannon’s index across all 12 samples and boxplots across the different land-uses (crop, reforested, agroforestry, uncultivated), for both fungal and bacterial species. The ecological indices were computed using custom Python (v3.12.4) code, while the plots were created using the seaborn (v0.13.2) and matplotlib (v3.8.4) libraries.

To complement diversity and abundance-based metrics, we also assessed the overlap of microbial taxa across land-use types using UpSet plots. ^24^ These diagrams summarize taxa overlap among land uses: horizontal bars indicate the total taxa per land use, and vertical bars indicate the number of taxa in each intersection defined by the filled-dot combinations. Single-dot intersections represent land-use–unique taxa, whereas multi-dot intersections represent shared taxa. A taxon was considered present in a specific land-use type if it was found in one or more replicates. These plots visualize the intersections of species sets and were created using the UpSetPlot (v0.9.0) package in Python.

### Statistical analysis

To assess how soil communities and environmental parameters vary across different land-use types, we performed comparative analyses focusing on diversity metrics, taxonomic composition, and abiotic factors. The 12 soil samples were grouped according to their land-use classification: crop, reforested, agroforestry, and uncultivated (3 replicates per group).

Richness and Shannon diversity values and relative abundances of fungal and bacterial species were compared across land-use types using boxplots. Statistical differences were assessed via one-way ANOVA followed by Tukey’s Honest Significant Difference (HSD) post hoc test. ^25^ Significance was defined at *p <* 0.05. Given the large number of species detected, only those exhibiting statistically significant differences between land-use types were visualized. Assumptions for parametric testing were assessed by applying the Shapiro–Wilk test for normality and Levene’s test for homogeneity of variance across land-use types. We note that, given the small number of biological replicates (n = 3 per group), these diagnostics must be interpreted cautiously.

Abiotic soil parameters were compared across land-use types using the same procedure. Electrical conductivity (EC) and boron (B) included left-censored observations (reported as *<*0.7 *μ*S/cm and *<* 0.5 mg/kg, respectively). These values were set at one-half the respective detection limit for the analysis. Again, we represented only the parameters where significant differences were found. All visualizations and tests were implemented using custom Python scripts with seaborn (v0.13.2), scipy (1.12.0), and statannotations (v0.7.1).

### Correlation analysis

First, we assessed samples similarity computing pairwise Pearson correlation matrices among the 12 soil samples using three different variable sets: bacterial species abundances, fungal species abundances, and abiotic parameters. These correlation matrices were visualized as heatmaps paired with hierarchical clustering.

Moreover, to identify and visualize the strongest associations among abiotic soil parameters, we computed pairwise Pearson correlation coefficients using the full set of measured variables for each one of the four land-uses types. To reduce visual clutter, we implemented a filtering strategy to retain only the most strongly correlated variable pairs.

Specifically, we first computed the Pearson correlation matrix across all abiotic parameters, using the absolute values of the coefficients. We then ranked all unique variable pairs by the magnitude of their correlation coefficients and selected the top 20 strongest associations (excluding self-correlations). The set of variables involved in these top correlations was extracted, and a reduced correlation matrix was constructed using only this subset. This filtered matrix was visualized as a heatmap with hierarchical clustering.

This analysis was carried out using custom Python (v3.12.4) scripts, while the heatmaps were created using seaborn (v0.13.2) package.

### Multivariate analysis

Before performing Multivariate Analysis, all variables were scaled to zero mean and unit variance using the StandardScaler function from the sklearn (v1.4.2) package. To reduce the dimensionality of the abiotic dataset and identify environmental gradients, we applied Principal Component Analysis (PCA) to the standardized abiotic parameters using the PCA function from sklearn (v1.4.2). The first two principal components were used to visualize the samples in reduced space, with samples colored by land-use type. Convex hulls were drawn around groups to highlight clustering by land use using the ConvexHulls function from scipy (v1.12.0).

To investigate the relationships between soil community composition and abiotic variables, we performed Redundancy Analysis (RDA) using species-level abundance data for fungi and bacteria as response matrices and a selected subset of abiotic parameters as explanatory variables. Because the number of abiotic variables exceeded the number of samples, we retained as explanatory variables only a selection of abiotic parameters. We first selected candidate predictors based on their contribution to the main abiotic gradient captured by PCA, retaining variables with an absolute loading >0.25 on the first principal component (PC1). To limit redundancy among predictors, we then assessed pairwise correlations among the selected abiotic variables and removed highly collinear variables using a correlation threshold of |r| = 0.9. The final set of abiotic predictors used in the RDA was: Cation Exchange Capacity (CEC), Boron (mg/kg), Exchangeable Calcium, Organic Matter, and Exchangeable Magnesium (meq/100 g).

RDA was performed using the rda function from the scikit-bio (v0.6.3) package. We visualised the results with a scatter plot in the 2-dimensional space defined by the main components of the RDA using matplotlib (v3.8.4).

### Functional analysis

Raw paired-end FASTQ files were reprocessed in QIIME 2 ^26^ (qiime2-amplicon-2025.7 version) to generate a PICRUSt2-compatible amplicon sequence variant (ASV) table together with the corresponding representative-sequence FASTA. Denoising, was performed with q2-dada2 (v2024.10.0) using denoise-paired. The resulting amplicon sequence variant (ASV) feature table and representative sequences were used to predict functional profiles with PICRUSt2 ^27^ (v2.4.1). In particular, we predicted Kyoto Encyclopedia of Genes and Genomes (KEGG).

Orthology (KO) terms, Enzyme Commission (EC) numbers, and MetaCyc metabolic pathways for each sample. These functional profiles were aggregated by land-use type; for each functional profile, we generated stacked bar plots showing the relative abundance of the 15 most prevalent functions across land uses. In addition, centered log-ratio (CLR) transformation was applied to each table (after compositional normalization), and principal component analysis (PCA) was performed to visualise patterns of functional differentiation among the four land-use categories. These exploratory analyses were conducted separately for KO, EC, and pathway profiles to compare the degree and structure of functional variation associated with land use. We note that the ASV table resulting from this reprocessing step may differ from the feature table produced by the Illumina pipeline. This approach was adopted because representative sequences were not available from the Illumina pipeline outputs, preventing direct use of that table in PICRUSt2. We note that functional profiles obtained with PICRUSt2 are inferred by reference-genome mapping and phylogenetic placement rather than by direct measurement of functional genes; for this reason, these results should be interpreted cautiously as predictive, also in light of the fact that PICRUSt2 used the QIIME 2-derived ASV table, which may differ from the Illumina pipeline feature table used for other statistical analyses.

## Discussion

In the present study we investigated, in typical northern Italy peri-urban agricultural landscapes, how land use is able to influence soil microbial communities, abiotic soil parameters, and functional potentials. This kind of study is important to identify the best agroecological practice in peri-urban area able to preserve a healthy soil. In fact, it is known that the expanding urbanization affects biodiversity and agrobiodiversity, for example in a recent paper was discussed the complex relationship between these aspects also focusing to urban and peri-urban land for the best use in terms of biodiversity. ^28^

Agroecological practices, included within the framework of Nature-Based Farming Solutions (e.g., landscape features, agroforestry), have the potential to activate and implement processes of diversification, ecological regeneration and multifunctional re-functionalization of rural areas. ^29^ In particular, agroforestry management, understood broadly as the management of productive and non-productive tree and shrub components, both within fields and along boundaries, such as hedgerows, tree lines, buffer strips, and small wooded patches, ^30^ is distinctive in its ability to interact with different levels of agroecosystem organization. ^31^

Our results collectively suggest that agroforestry systems compared to uncultivated, reforested and crop land use, can play a pivotal role in restoring soil health and enhancing EC, reinforcing their importance as nature-based solutions for sustainable land management. Across the four peri-urban land uses, we observed consistent land-use–associated differences in both abiotic soil properties and microbial community structure, supporting the view that land management is a key determinant of soil health indicators in this landscape. In fact, the observed differences in fungal and bacterial community composition across land-use types confirm that land management has a measurable impact on soil microbiota. Agroforestry soils were characterized by the highest number of unique taxa, whereas reforested soils showed the lowest richness and diversity, aligning with previous studies reporting that microbial communities in early-stage reforested soils are typically less diverse and more compositionally simplified than in later successional stages. ^32^ ^–^ ^34^

In a recent meta-analysis where was investigated the effects of agroforestry on ES provision in Europe, ^35^ was reported that, compared to conventional land uses such as pastures, arable crops, or forests, agroforestry supports higher levels of biodiversity and ecosystem goods and services. In this connection, the study by Beillouin *et al*. ^36^ confirmed the capacity of agroforestry to significantly influence multiple ES, including biodiversity, production, water regulation and quality, pest and disease control, and soil quality.

Furthermore, we found that fungal communities had a stronger variation in dominant genera across land uses in comparison to bacterial communities. The predominance of *Mortierella*, *Fusarium*, and *Trebouxia* in fungal communities, as well as the dominance of Acidobacteria and Gemmatimonadetes in bacterial communities, suggests that these groups may respond to management-driven gradients in soil resources and disturbance. The high abundance of Mortierella is of particular interest, as some studies have indicated that Mortierella is associated with disease-suppressive soils, which goes with the accentuated complexity of undisturbed and diversified systems such as Silvoarable Agroforestry Systems (SAF). ^37^ ^–^ ^39^ These symbioses are crucial for the nutrient cycle in forests and can play an important role in ecological succession processes. Considering the richness, we found the highest levels for crops and agroforestry for both bacteria and fungi. All together these analysis pointed out the deep impact of land use on the soil microbiome.

We then investigated how abiotic parameters such as for example cation-exchange capacity (CEC), total nitrogen, and boron, affect microbial communities. Multivariate analyses (PCA and RDA) revealed that abiotic parameters were key determinants of microbial community structure. This finding supports the hypothesis that soil fertility parameters not only reflect the chemical status of soils but also shape microbial niches and resource availability. Interestingly, agroforestry and uncultivated soils exhibited more heterogeneous clustering patterns of abiotic variables, suggesting a higher degree of spatial complexity and potentially greater resilience to disturbances. Functional predictions using PICRUSt2 indicated that while a core set of metabolic functions was shared across all land uses, agroforestry soils exhibited a broader functional repertoire. This functional diversification is consistent with the idea that agroforestry systems enhance ecological functions beyond productivity by providing microhabitats and diversified root exudates, as well as creating microhabitats that support a wider range of microbial guilds, thereby improving nutrient cycling and soil multifunctionality. ^40^ ^,^ ^41^ Our results provide evidence that management practices can influence not only who is present in the soil microbiome but also what functions they are capable of performing. This is aligned with the growing body of literature advocating agroforestry as a key tool in the ecological refunctionalization of rural and peri-urban landscapes. ^42^ ^,^ ^43^ By promoting biodiversity, improving soil structure, and enhancing carbon sequestration, agroforestry systems directly contribute to several Sustainable Development Goals (SDGs), including Zero Hunger (SDG 2), Climate Action (SDG 13), and Life on Land (SDG 15). The ES provided by agroforestry-managed soils include: soil conservation; soil health; carbon sequestration; nutrient cycling and enrichment; and the sequestration, transformation, and detoxification of chemicals into non-toxic forms. These benefits are difficult to measure independently, as they are interconnected. For example, increased carbon sequestration enhances soil nutrient status by improving cation exchange capacity, soil stability, and soil quality, while also reducing soil erosion. ^44^

Overall, our study highlights the need for composite soil health indicators that integrate microbial, chemical, and physical parameters, as recommended by international frameworks such as the FAO’s Global Soil Partnership and the EU Soil Strategy for 2030. Despite the robustness of our findings, the limited number of replicates (n = 3 per land-use type) constrains the statistical power of some comparisons, particularly for fungal communities where natural variability can be high. Future research should include temporal sampling to capture seasonal dynamics and longitudinal changes following management interventions.

## Conclusion

Our findings show that land-use type has a significant impact on soil microbial communities, with agroforestry emerging as a promising agroecological practice. By increasing microbial diversity, supporting unique taxa, and broadening functional profiles, agroforestry can enhance ecosystem services, including nutrient cycling, carbon sequestration, and resilience to environmental stress. These findings support the incorporation of agroforestry into peri-urban agricultural landscapes as a strategy to balance food production with biodiversity conservation and climate regulation.

From a policy standpoint, this study emphasizes the importance of soil health indicators that incorporate biological aspects, as recommended by the FAO and the EU Soil Strategy 2030. Agroecological methods should be prioritized in land management frameworks to enhance soil ecosystem services and global health.

## Ethics and consent

Ethical approval and consent were not required.

## Data availability

UNIMI Dataverse. Replication Data for: Soil Health and Microbial Diversity Across Land-Use Types: Evidence for Agroecological Management in Peri-Urban Areas. https://dataverse.unimi.it/dataset.xhtml?persistentId=doi:10.13130/RD_UNIMI/ZGZY4Q ^45^

This project contains the following underlying data:

16 s Genus Level Aggregate Counts 2.csv: ITS OTU table at genus level ITS Species Level Aggregate Counts.csv: ITS OTU table at species level 16 s Species Level Aggregate Counts.csv: 16 s OTU table at species level ITS Genus Level Aggregate Counts.csv: 16 s OTU table at genus level Samples.xlsx: metadata specifying the type of soil for each sample.

Data is available under the terms of the CC BY 4.0 licence.

## References

[1] Millennium Ecosystem Assessment: Ecosystems and human well-being: wetlands and water synthesis.2005. Reference Source

[2] Stoate C Arau’jo M Borralho R : Conservation of European farmland birds: abundance and species diversity. *Ornis Hung.* 2003;12(13):33–40. Reference Source

[3] Gliessman SR Rosado-May FJ Guadarrama-Zugasti C : Agroecología: promoviendo una transición hacia la sostenibilidad. *Ecosistemas.* 2007;16(1):13–23. Reference Source

[4] Ingegnoli V : *Landscape bionomics biological-integrated landscape ecology* : Springer,2015. 10.1007/978-88-470-5226-0

[5] Borrelli P Robinson DA Fleischer LR : An assessment of the global impact of 21st century land use change on soil erosion. *Nat Commun.* 2017;8(1):2013. 10.1038/s41467-017-02142-7 29222506 PMC5722879

[6] Chiaffarelli G Vagge I : Cities vs countryside: an example of a science-based peri-urban landscape features rehabilitation in Milan (Italy). *Urban For Urban Green.* 2023;86:128002. 10.1016/j.ufug.2023.128002

[7] Norman C Surjan A Booth M : Making resilience a reality: the contribution of peri-urban ecosystem services (BGI) to urban resilience. In: *Ecosystem-Based disaster and climate resilience: integration of blue-green infrastructure in sustainable development* . Springer,2021;185–200. 10.1007/978-981-16-4815-1_8

[8] Colucci A : Peri-urban/peri-rural areas: identities, values and strategies. In: *Peri-Urban areas and food-energy-water nexus: sustainability and resilience strategies in the age of climate change* . Springer,2016;99–104. 10.1007/978-3-319-41022-7_12

[9] Butt A : Sustainable, resilient, regenerative? The potential of melbourne’s peri-urban region. *Front Sustain Cities.* 2024;6:1391712. 10.3389/frsc.2024.1391712

[10] Dal Borgo AG Bocchi S Capocefalo V : Agroforestry for the city: farmscaping the urban fringe through transformative and participatory action research in Milan. *Agroforest Syst.* 2025;99(5):125. 10.1007/s10457-025-01198-5

[11] Dal Borgo AG Chiaffarelli G Capocefalo V : Agroforestry as a driver for the provisioning of peri-urban socio-ecological functions: a trans-disciplinary approach. *Sustainability.* 2023;15(14):11020. 10.3390/su151411020

[12] Erdogan HE Havlicek E Dazzi C : Soil conservation and Sustainable Development Goals (SDGs) achievement in europe and central asia: which role for the European soil partnership? *International Soil and Water Conservation Research.* 2021;9(3):360–369. 10.1016/j.iswcr.2021.02.003

[13] Heuser I : Soil governance in current European Union law and in the European green deal. *Soil Secur.* 2022;6:100053. 10.1016/j.soisec.2022.100053

[14] Panagos P Montanarella L Barbero M : Soil priorities in the European Union. *Geoderma Reg.* 2022;29:e00510. 10.1016/j.geodrs.2022.e00510

[15] Pereira P Bogunovic I Muñoz-Rojas M : Soil ecosystem services, sustainability, valuation and management. *Curr Opin Environ Sci Health.* 2018;5:7–13. 10.1016/j.coesh.2017.12.003

[16] De Clercq P De Vroe A Janssens P : Effect of a soil water balance controlled irrigation on the cultivation of *Acer pseudoplatanus* forest Tree liners under non-limiting and limiting soil water conditions. *Horticulturae.* 2025;11(4):435. 10.3390/horticulturae11040435

[17] Khan A Rao TS : Molecular evolution of xenobiotic degrading genes and mobile DNA elements in soil bacteria. In: *Microbial Diversity in the Genomic Era* Elsevier,2019;657–678. 10.1016/B978-0-12-814849-5.00036-8

[18] Longo A : Openagri – 18 progetti x 30 ettari: un masterplan per un parco della sperimentazione agroecologica. Workshop working document (wp7), Dipartimento DAStU, Politecnico di Milano; Dipartimento ABC Politecnico di Milano; Dipartimento ESP Universit’a degli Studi di Milano, Milano, Italia. Nell’ambito del progetto OpenAgri, bando europeo Urban Innovative Actions (UIA),2018.

[19] LIAISON Consortium : Milano porta verde — European Rural Innovation Ambassador Horizon 2020 project LIAISON, Grant Agreement No. 773418,2020. Reference Source

[20] Prusicki M : Area sud-milano. Uno scenario strategico di riqualificazione paesistica del basso milanese. In: *LOTO Landscape Opportunities. La Gestione Paesistica delle Trasformazioni Territoriali. Complessit’a Territoriale e Valorizzazione del Paesaggio.* Esperienze a Confronto in Lombardia, Regione Lombardia,2006;52–92.

[21] Costantini E Dazzi C :1999; *World Reference Base for Soil Resources. Base di riferimento mondiale per le risorse pedologiche* : CRA-ABP. Reference Source

[22] ERSAF : Regional soil database Losan.2008. Reference Source

[23] Klindworth A Pruesse E Schweer T : Evaluation of general 16s ribosomal RNA gene PCR primers for classical and next-generation sequencing-based diversity studies. *Nucleic Acids Res.* 2013;41(1):e1. 10.1093/nar/gks808 22933715 PMC3592464

[24] Lex A Gehlenborg N Strobelt H : Upset: visualization of intersecting sets. *IEEE Trans Vis Comput Graph.* 2014;20(12):1983–1992. 10.1109/TVCG.2014.2346248 26356912 PMC4720993

[25] Tukey JW : Comparing individual means in the analysis of variance. *Biometrics.* 1949;5(2):99–114. 10.2307/3001913 18151955

[26] Bolyen E Rideout JR Dillon MR : Reproducible, interactive, scalable and extensible microbiome data science using QIIME 2. *Nat Biotechnol.* 2019;37(8):852–857. 10.1038/s41587-019-0209-9 31341288 PMC7015180

[27] Douglas GM Maffei VJ Zaneveld JR : PICRUSt2 for prediction of metagenome functions. *Nat Biotechnol.* 2020;38(6):685–688. 10.1038/s41587-020-0548-6 32483366 PMC7365738

[28] Zimmerer KS Duvall CS Jaenicke EC : Urbanization and agrobiodiversity: leveraging a key nexus for sustainable development. *One Earth.* 2021;4(11):1557–1568. 10.1016/j.oneear.2021.10.012

[29] Wezel A Goris M Bruil J : Challenges and action points to amplify agroecology in Europe. *Sustainability.* 2018;10(5):1598. 10.3390/su10051598

[30] Santiago-Freijanes JJ Mosquera-Losada MR Rois-Díaz M : Global and European policies to foster agricultural sustainability: agroforestry. *Agroforest Syst.* 2021;95(5):775–790. 10.1007/s10457-018-0215-9

[31] Montagnini F Fierro S : Functions of agroforestry systems as biodiversity Islands in productive landscapes. In: *Biodiversity Islands: Strategies for Conservation in Human-dominated Environments* . Springer,2022;89–116. 10.1007/978-3-030-92234-4_4

[32] Kong W Wei X Wu Y : Afforestation can lower microbial diversity and functionality in deep soil layers in a semiarid region. *Glob Chang Biol.* 2022;28(20):6086–6101. 10.1111/gcb.16334 35808859

[33] Beule L Vaupel A Moran-Rodas VE : Abundance, diversity, and function of soil microorganisms in temperate alley-cropping agroforestry systems: a review. *Microorganisms.* 2022;10(3):616. 10.3390/microorganisms10030616 35336196 PMC8953468

[34] De Clerck C Andrianarisoa KS Lassois L : Silvoarable agroforestry systems as promising practices for improving soil biological health: recent advances and future challenges in Western European region. *Soil Use Manage.* 2025;41(3):e70104. 10.1111/sum.70104

[35] Torralba M Fagerholm N Burgess PJ : Do European agroforestry systems enhance biodiversity and ecosystem services? A meta-analysis. *Agriculture, ecosystems & environment.* 2016;230:150–161. 10.1016/j.agee.2016.06.002

[36] Beillouin D Ben-Ari T Malézieux E : Positive but variable effects of crop diversification on biodiversity and ecosystem services. *Glob Chang Biol.* 2021;27(19):4697–4710. 10.1111/gcb.15747 34114719

[37] Siles JA Vera A Díaz-López M : Land-use-and climate-mediated variations in soil bacterial and fungal biomass across Europe and their driving factors. *Geoderma.* 2023;434:116474. 10.1016/j.geoderma.2023.116474

[38] Kim HS Lee SH Jo HY : Diversity and composition of soil *Acidobacteria* and *Proteobacteria* communities as a bacterial indicator of past land-use change from forest to farmland. *Sci Total Environ.* 2021;797:148944. 10.1016/j.scitotenv.2021.148944 34298360

[39] Gossner MM Lewinsohn TM Kahl T : Land-use intensification causes multitrophic homogenization of grassland communities. *Nature.* 2016;540(7632):266–269. 10.1038/nature20575 27919075

[40] Ullah S Han X Ali I : Ecological impacts of diversified agroforestry on soil nutrients and bacterial communities in *pinus massoniana* plantations in the southern subtropics. *Industrial Crops and Products.* 2024;222(Part 4):119933. 10.1016/j.indcrop.2024.119933

[41] Soliveres S Van Der Plas F Manning P : Biodiversity at multiple trophic levels is needed for ecosystem multifunctionality. *Nature.* 2016;536(7617):456–459. 10.1038/nature19092 27533038

[42] Castle SE Miller DC Merten N : Evidence for the impacts of agroforestry on ecosystem services and human well-being in high-income countries: a systematic map. *Environ Evid.* 2022;11(1):10. 10.1186/s13750-022-00260-4 39294716 PMC11378871

[43] Jose S : Agroforestry for ecosystem services and environmental benefits: an overview. *Agroforest Syst.* 2009;76(1):1–10. 10.1007/s10457-009-9229-7

[44] Udawatta RP Rankoth LM Jose S : Agroforestry and biodiversity. *Sustainability.* 2019;11(10):2879. 10.3390/su11102879

[45] Replication Data For: Soil Health and Microbial Diversity Across Land-Use Types: Evidence for Agroecological Management in Peri-Urban Areas. 10.13130/RD_UNIMI/ZGZY4Q

