# Supplementary material for: Soil health and microbial diversity across land-use types: Evidence for agroecological management in peri-urban areas
Source: Open Res Eur. 2026 Aug 22;6:29. Originally published 2026 Jan 26. [Version 4] doi: 10.12688/openreseurope.22066.4 (PMC13555044; doi:10.12688/openreseurope.22066.4)
Supplement: Peer review history

# Reviewer report: Reviewer response for version 4

**DOI:** 10.21956/openreseurope.26886.r79576

**Published:** 2026-09-10

**Authors:** Naba Raj Amgain

**Affiliations:** 1 Purdue University, West Lafayette, Indiana, USA

Comments to the authors: The authors have addressed all comments and made the requested revisions. The changes are appropriate and I have no further concerns and recommend acceptance of the manuscript.

Is the study design appropriate and does the work have academic merit?

Yes

Is the work clearly and accurately presented and does it cite the current literature?

Yes

If applicable, is the statistical analysis and its interpretation appropriate?

No

Are all the source data underlying the results available to ensure full reproducibility?

Yes

Are the conclusions drawn adequately supported by the results?

Yes

Are sufficient details of methods and analysis provided to allow replication by others?

No

Reviewer Expertise:

NA

I confirm that I have read this submission and believe that I have an appropriate level of expertise to confirm that it is of an acceptable scientific standard.

# Reviewer report: Reviewer response for version 3

**DOI:** 10.21956/openreseurope.26178.r76362

**Published:** 2026-07-15

**Authors:** Naba Raj Amgain

**Affiliations:** 1 Purdue University, West Lafayette, Indiana, USA

This study evaluates the effect of agroecological land management in peri-urban areas on microbial diversity and soil health parameters. Overall, the manuscript is well organized, clearly written, and easy to follow.

 Specific comments are listed below:

**Results:**

 Please review the opening sentence of each results subsection. Any sentence that describes the methodology should be removed, as the experimental procedures have already been presented in the Methods section. Where an introductory sentence is necessary, it should be revised to provide context for the results without repeating methods used.

 Delete “To characterize difference………………. occurrence among land uses”. This is the repetition of materials and methods. - Page 5

 Delete” In order to assess…………we performed ordination analysis.” – page 9

 Provide the abbreviations as footnotes in each corresponding figure.

**Methods**

 The methods section describes the sequencing and data analysis procedures; however, details of the methods used to measure soil physical parameters are missing. Please include a detailed description of how the soil samples were processed and analyzed for all reported soil physical parameters.

 “The study is applied to an agricultural….” Change to “The study was applied/conducted to an agricultural….”

 Change “The study area covers……………...” to “The study area covered………….?

 Materials methods should be written in past tense.

**Statistical Analysis**

 Most of the content currently included under the statistical analysis section belongs to the methods section. This section should only describe the statistical methods and software used. For example, include statements such as: "Statistical differences were assessed using one-way analysis of variance (ANOVA), with significance declared at *p* < 0.05."

**Discussion**

 Consider moving the first two paragraphs to the end of the discussion section, as they contain concluding remarks and discuss the study's limitations. Placing these paragraphs at the end would improve the logical flow of the discussion by allowing the findings to be interpreted before presenting the overall conclusions and limitations.

 Rewrite sentences beginning with "We …...." (e.g., “we then investigated….”- page 20)- please check this throughout the manuscript. The use of first-person pronouns (e.g., 'we') should be minimized and revised where necessary

 Suggest changing “In a recent meta-analysis where was investigated……………” to “In a recent meta-analysis that investigated…….”

 Delete “We then investigated how abiotic………………...affect microbial communities”

Is the study design appropriate and does the work have academic merit?

Yes

Is the work clearly and accurately presented and does it cite the current literature?

Yes

If applicable, is the statistical analysis and its interpretation appropriate?

No

Are all the source data underlying the results available to ensure full reproducibility?

Yes

Are the conclusions drawn adequately supported by the results?

Yes

Are sufficient details of methods and analysis provided to allow replication by others?

No

Reviewer Expertise:

soil health

I confirm that I have read this submission and believe that I have an appropriate level of expertise to confirm that it is of an acceptable scientific standard, however I have significant reservations, as outlined above.

# Response

**Published:** 2026-08-04

**Authors:** Caterina La Porta

**Affiliations:** Universita degli Studi di Milano, Milan, Lombardy, Italy

**Response to Reviewers** **Manuscript title:**  Soil health and microbial diversity across land-use types: Evidence for agroecological management in peri-urban areas  

**Reviewer #3:** This study evaluates the effect of agroecological land management in peri-urban areas on microbial diversity and soil health parameters. Overall, the manuscript is well organized, clearly written, and easy to follow.

***Answer:*** We thank the reviewer for the careful reading of our manuscript and for the positive overall assessment. We address the comments point by point below; we believe they have improved the clarity and rigour of the revised manuscript.

 Specific comments are listed below:

**Results:**

 Review 3: Please review the opening sentence of each results subsection. Any sentence that describes the methodology should be removed, as the experimental procedures have already been presented in the Methods section. Where an introductory sentence is necessary, it should be revised to provide context for the results without repeating methods used.

***Answer:*** We thank the Reviewer for the suggestion. We have revised the opening sentence of each Results subsection and removed those that merely restated the methodology already presented in the Methods.

 Review 3: Delete “To characterize difference………………. occurrence among land uses”. This is the repetition of materials and methods. - Page 5

 ​​​​​​​ ***Answer: *** The introductory sentence was removed.

 Review 3: Delete” In order to assess…………we performed ordination analysis.” – page 9

 ​​​​​​​ ***Answer: *** The introductory sentence was removed.

 Review 3: Provide the abbreviations as footnotes in each corresponding figure.

 ​​​​​​​ ***Answer:*** We have added footnotes defining the abbreviations used in each corresponding figure. Abbreviation keys were added to the captions of Figures 7, 8, 9, and 10; full parameter names and units also remain available in Table 2.

**Methods**

 Review 3: The methods section describes the sequencing and data analysis procedures; however, details of the methods used to measure soil physical parameters are missing. Please include a detailed description of how the soil samples were processed and analyzed for all reported soil physical parameters.

 ​​​​​​​ ***Answer: *** As suggested, we have added a description of the analytical methods used for the reported soil parameters. Table 2 now includes an additional column specifying the method applied to the parameters, as reported by the laboratory that performed the analyses.

 Review 3: “The study is applied to an agricultural….” Change to “The study was applied/conducted to an agricultural….”

 Change “The study area covers……………...” to “The study area covered………….?

 Materials methods should be written in past tense.

 ​​​​​​​ ***Answer:*** Corrected

**Statistical Analysis**

 Review 3: Most of the content currently included under the statistical analysis section belongs to the methods section. This section should only describe the statistical methods and software used. For example, include statements such as: "Statistical differences were assessed using one-way analysis of variance (ANOVA), with significance declared at *p* < 0.05."

 ​​​​​​​ ***Answer:*** We thank the reviewer for the suggestion; we have reorganized this part of the Methods. The former section has been renamed "Data analysis" and now contains the data-processing, diversity-computation, ordination, and functional-prediction procedures. A separate "Statistical analysis" section has been added that describes only the statistical tests and software.

**Discussion**

 Review 3: Consider moving the first two paragraphs to the end of the discussion section, as they contain concluding remarks and discuss the study's limitations. Placing these paragraphs at the end would improve the logical flow of the discussion by allowing the findings to be interpreted before presenting the overall conclusions and limitations.

 ​​​​​​​ ***Answer: *** As suggested, we have moved the two opening paragraphs of the Discussion to the end of the Discussion. A brief statement of the study's aim was retained at the start to introduce the section.

 Review 3: Rewrite sentences beginning with "We …...." (e.g., “we then investigated….”- page 20)- please check this throughout the manuscript. The use of first-person pronouns (e.g., 'we') should be minimized and revised where necessary

 ​​​​​​​ ***Answer:***  As suggested, we have reduced the use of first-person pronouns throughout the Discussion.

 Review 3: Suggest changing “In a recent meta-analysis where was investigated……………” to “In a recent meta-analysis that investigated…….”   

 ​​​​​​​ ***Answer: *** Corrected

 Review 3: Delete “We then investigated how abiotic………………...affect microbial communities”

 ​​​​​​​ ***Answer:*** We have removed this sentence.

# Reviewer report: Reviewer response for version 1

**DOI:** 10.21956/openreseurope.23877.r69473

**Published:** 2026-02-25

**Authors:** Carolyn Cornell

**Affiliations:** 1 Colorado State University, Boulder, Colorado, USA

In the manuscript “Soil health and microbial diversity across land-use types: Evidence of agroecological management in peri-urban areas”, the authors aimed to determine how land-use type, and therefore the associated management, impacted soil health using “different indicators” across peri-urban landscapes typical to northern Italy, including a crop field, uncultivated area, reforested land, and agroforestry site. Three soil samples were collected per site during a single collection to examine abiotic soil properties, and bacterial and fungal communities using amplicon sequencing. Based on the study, the authors concluded that land-use type significantly impacts soil microbial communities and that agroforestry may help increase or preserve diversity in agricultural land. The authors also said that the study provides mechanistic insight into how land management shapes biodiversity and informs strategies for improving overall soil health.

 While the authors conducted an interesting study, it was difficult to assess the robustness due to insufficient detail in the materials and methods, especially regarding sequencing depth and data processing. The sample size also limits the study's power. There were also several conclusions drawn on the impact of abiotic properties and soil microbial communities, with few actual correlations done between the two datasets. The number of figures could also be reduced, with many showing very similar information. Additionally, the introduction set the manuscript up to focus on soil health, yet the results did not necessarily follow that theme. Pointing out more on the differences between sites that would support that a certain land-use type may be “healthier” because you found, for example, more beneficial soil taxa or increased carbon/OM compared to more managed lands, would help better frame the story.

**Comments by section:**

**Abstract**

- P. 1: Suggest changing “—such as” in the methods part of the abstract to “including” since that is the full list of land-use types used, not just several examples.
- P. 2: Evenness is mentioned in the results section of the abstract, while the conclusion drawn makes sense, it would be better to actually calculate evenness as one of your alpha-diversity metrics.  
- P. 2: In the results section of the abstract, while it was found that the abiotic factors pH, nitrogen, and CEC differed with land-use type, no correlations were made to the microbial communities. It was almost done with the RDA, but the vectors showing the abiotic factors were not included in the plot.
- P. 2: I would change the word “mechanistic” as the study did not show a mechanism but an association or correlation.
- P. 2: Suggest changing “resilience” to “potential resilience” as this was not examined in this study.

**Introduction:**

- P. 4: The last sentence of the first paragraph in the introduction is too long, making it hard to follow. Break it up into two sentences.  
- P. 4: In this sentence, “When considering the subset of peri-urban agricultural systems, these processes are further exacerbated.” I suggest moving the peri-urban fringe definition to the beginning of the paragraph, closer to where this term is introduced.
- P. 4: Need to define “soil health” as the definition is not always consistent. It also quickly moves on to the importance of soil health as a whole, but it would help to mention its particular relevance to peri-urban areas, which was the topic of the previous paragraph.
- P. 4: “According to 16,..” Should be changed to “author’s name and colleagues” followed by the citation.
- P. 4: “contest” needs to be changed to “context” in the last paragraph of the introduction.
- P. 4: The description of the study could be improved in the last paragraph. These “different indicators” need to be explained more, especially if the authors are suggesting they can be used in other studies as indicators of soil health.

**Methods:**

- The overall organization of the data analysis of the methods is a bit confusing. It makes more sense to break your methods into sequence analysis and statistical analysis, rather than a section for each test.
- I do not understand the reasoning behind using the “Illumina 16S Metagenomics pipeline” in one section, then, in another section, saying the data was processed again using “QIIME 2.” Only one should be used as they will produce different OTU/ASV tables. No link or reference to the Illumina pipeline was provided, so I was unable to see how the methods compared.
- Why not perform beta-diversity analysis? This would be a better comparison of diversity differences across sites as it takes into account the community structure/taxa at each site.
- The methods need to be checked for tense usage. Often using the present tense when needing to use the past tense when describing tests and sampling.
- P. 11: Add a sentence making it clear that the management history is presented in Table 1. I was looking for it in the text because it wasn’t clear that I could find it in the table.
- P. 11: Confused on why four quadrants were established and one sampling point was established in each, but only 3 samples were taken.
- P. 11: When were the samples collected? Include the year and the month at least. This is important since it is said some land uses were established in 2019, so it is helpful to know how long since the establishment of the site where samples were taken.
- P. 14: There is no information given on how soil parameters were measured. Either cite other papers that have this information, or, if they were sent somewhere, include the location and the link so readers can find the methods there.
- P. 14: Change “DNA quality control checks” to “DNA quality and quantity were checked” since the Nanodrop is generally for quality and Qubit for quantity.
- P. 14: Add the specific 16S primers (e.g., 314F and 806R).
- P. 14: Add a link or citation where the specific PCR protocol can be found. I also recommended including a short summary, such as whether it was done in replicates, in two steps, etc.
- P. 14: Add a link or citation for the “16S Metagenomic pipeline”. Was there a different pipeline for the ITS, or was it adapted at all for differences between ITS and 16S reads?
- P. 14: The database versions used are dated. Ideally, should use more recent releases of both databases.
- P. 14: It is mentioned that negative controls were included. Were any sequences found in the negative controls, and if so, include how they were accounted for in the data.
- P. 14: While using relative abundance normalization for the data is fine, information should be included on the average sequencing depth for each dataset, and if there was a cutoff on the minimum number of sequences considered usable for a sample. Given soil diversity, sequencing depth can be important.
- P. 15: It should be mentioned that the metrics used are both alpha-diversity metrics.
- P. 15: Does the “16S Metagenomic pipeline” produce OTUs or ASVs? It is generally just referred to as species-level assignments in the methods.
- P. 16: For the UpSet plots, was a taxa considered to be present in both land uses if it was present in just one of the replicates or more?
- P. 16: Was the data checked for normality and homogeneity of variance before determining that a parametric test (ANOVA) was the right choice? If so, include that in the methods.
- P. 18: Was there a correlation coefficient cutoff value to be considered “strongly” correlated?
- P. 19: Did you consider the correlation between the abiotic factors when doing your RDA? Also, need to add the vectors of the abiotic factors to the RDA so readers can see which factors were associated with each land-use type.
- P. 19: Please add a statement about the use of PICRUSt, such as, “however, these predictions are based on reference genomes and do not represent direct measurements of functional genes. Therefore, results should be interpreted cautiously,” considering its limitations.

**Results**

- For the results, my main comments would be to reduce the number of figures to make the message more concise. For example, the alpha-diversity metrics are shown by field with box plots (Fig. 2), then by replicate (Fig. 3). Unless a main point is within site variation, then Figure 3 is not needed. The same for showing heatmaps of the top 20 correlations between abiotic factors, then showing heatmaps with all variables.
- In the results text, there needs to be a stronger focus on the differences between land uses to convey that converting to certain land-use types may improve “soil health,” as seen by increases in diversity, richness, OM, etc., to match the story set out in the introduction.
- P. 4: When saying “significantly” in terms of results, it can be helpful to put the p-value in parentheses after it, especially since the method is after the results, where significance is defined. I recommended this for the whole results section.
- P. 4: “certain genera significantly more abundant in specific samples and in specific land-use types”, which ones especially on a land-use base comparisons?
- P. 4: The genera “Mortierella, Fusarium, and Trebouxia” need to be italicized.
- P. 4: I do not think this conclusion is supported because no differences in relative abundance were explained between sites in the text and no differences in organic inputs have been presented yet, “From an agroecological point of view, this results clearly demonstrate the role of agronomical management that enhance soil organic inputs (e.g., manure, green mulch, compost application, agroforestry litterfall) in sustaining fungal diversity.”
- P. 4-5: For the differences in alpha-diversity metrics, state if they were significant, not just noticeable. The statement about replicating following it makes it sound like you would have found significant values if you had more replicates, which is not necessarily true. Consider rewording or removing that statement.
- P. 5: Fungi is a kingdom, not a domain.
- P. 5-6: “Such diversification contributes to ecosystem resilience and multifunctionality, which are essential for effective biological pest control.” A statement like this is better for the discussion, so you can show that other studies have shown this. Otherwise, it appears to be an unsupported claim in the results.
- P. 6: Again, explain the differences in what taxa you saw that were significantly different between land uses. The figure captions also says genera instead of phyla. It would also make more sense to talk about the differences in phyla (Fig. 4/5) before talking about the genera (Fig. 1).
- P. 6: What is meant by “bacterial and fungal profiles”? Do you mean the special-level abundance tables? This information on what went into the tables needs to be included in the Figure 6 caption. Based on the coefficient gradient for your heatmap, I also cannot tell if it includes negative correlations. I number at the bottom of the gradient needs to be included.
- P. 7: The conclusions on soil stability based on abiotic variable correlation need to be moved to the discussion, so examples that support this can be cited. It would also be a good place to discuss how the management specific to the crop and reforested sites can lead to shared drivers of these properties.
- P. 8: Redundant/highly correlated variables can impact a PCA and RDA. It would be useful to include details in the figures' captions and in the methods section on how this was handled, since strong correlations between factors were shown in Figure 7.
- P. 8: Discussing the differences in abiotic factors between land uses (Fig. 13), which help show what factors shift under the different management and may help support the PCA results below, showing that there are differences in soil factors by land use.
- P. 8: I believe I mentioned this before, but all the abiotic vectors are in the RDA, so you can discuss which factors were associated with different land uses.
- P. 9: For the functional differences, again explain the important differences between land use. Did you see a loss or gain of a certain function with land use?

**Discussion**

- Overall, the discussion needs to focus more on how the results presented were caused by differences in land use and how the management practices associated with those land uses may have shaped them. This then needs to be brought back to the idea of soil health. Currently, it is not focusing enough on the differences between land uses, and again, it just says there were broad differences between land uses.

Altogether, the authors present an interesting study on how land use type may influence soil microbial diversity and overall soil health in a peri-urban landscape. However, significant revisions are needed to improve the clarity, robustness, and alignment between the stated objectives and the results. The authors should provide more detail in the Materials and Methods section on sequencing data and management practices for each land-use type. The number of figures should be reduced, and the order of figures reconsidered to group similar topics (e.g., keep all relative abundance differences together). The results and discussion should focus more on differences between land-use types, mentioning specific differences and how management practices influence soil properties and microbial communities to support claims about soil health. Lastly, moderate claims about mechanisms and resilience to better align with the data presented.

Is the study design appropriate and does the work have academic merit?

Yes

Is the work clearly and accurately presented and does it cite the current literature?

Partly

If applicable, is the statistical analysis and its interpretation appropriate?

Partly

Are all the source data underlying the results available to ensure full reproducibility?

Yes

Are the conclusions drawn adequately supported by the results?

Partly

Are sufficient details of methods and analysis provided to allow replication by others?

No

Reviewer Expertise:

Soil microbial ecology, nitrogen cycling, and agricultural systems

I confirm that I have read this submission and believe that I have an appropriate level of expertise to confirm that it is of an acceptable scientific standard, however I have significant reservations, as outlined above.

# Response

**Published:** 2026-03-25

**Authors:** Caterina La Porta

**Affiliations:** Universita degli Studi di Milano, Milan, Lombardy, Italy

**REVIEWER #2**

**Reviewer: **In the manuscript “Soil health and microbial diversity across land-use types: Evidence of agroecological management in peri-urban areas”, the authors aimed to determine how land-use type, and therefore the associated management, impacted soil health using “different indicators” across peri-urban landscapes typical to northern Italy, including a crop field, uncultivated area, reforested land, and agroforestry site. Three soil samples were collected per site during a single collection to examine abiotic soil properties, and bacterial and fungal communities using amplicon sequencing. Based on the study, the authors concluded that land-use type significantly impacts soil microbial communities and that agroforestry may help increase or preserve diversity in agricultural land. The authors also said that the study provides mechanistic insight into how land management shapes biodiversity and informs strategies for improving overall soil health.

 While the authors conducted an interesting study, it was difficult to assess the robustness due to insufficient detail in the materials and methods, especially regarding sequencing depth and data processing. The sample size also limits the study's power. There were also several conclusions drawn on the impact of abiotic properties and soil microbial communities, with few actual correlations done between the two datasets. The number of figures could also be reduced, with many showing very similar information. Additionally, the introduction set the manuscript up to focus on soil health, yet the results did not necessarily follow that theme. Pointing out more on the differences between sites that would support that a certain land-use type may be “healthier” because you found, for example, more beneficial soil taxa or increased carbon/OM compared to more managed lands, would help better frame the story.

**Answer:** We thank the review for her comments. We have now revised the manuscript accordingly.

**Comments by section:**

**Reviewer: Abstract** P. 1: Suggest changing “—such as” in the methods part of the abstract to “including” since that is the full list of land-use types used, not just several examples.

**Answer:** Done

**-----** **Reviewer:** Abstract P. 2: Evenness is mentioned in the results section of the abstract, while the conclusion drawn makes sense, it would be better to actually calculate evenness as one of your alpha-diversity metrics.

**Answer: **We changed accordingly

 ----- **Reviewer: **Abstract P. 2: In the results section of the abstract, while it was found that the abiotic factors pH, nitrogen, and CEC differed with land-use type, no correlations were made to the microbial communities. It was almost done with the RDA, but the vectors showing the abiotic factors were not included in the plot.

**Answer:** We agree with the referee. We revised the RDA analysis and the plots (Fig. 6) updated to include the abiotic vectors, so the associations between land-use clustering and the main abiotic gradients are now explicit.

**----------** **Reviewer**: Abstract P. 2: I would change the word “mechanistic” as the study did not show a mechanism but an association or correlation.

**Answer:** Changed accordingly.

 ----------- **Reviewer **Abstract P. 2: Suggest changing “resilience” to “potential resilience” as this was not examined in this study.

**Answer**: Done

 ---------------- **Reviewer: **Introduction **:** P. 4: The last sentence of the first paragraph in the introduction is too long, making it hard to follow. Break it up into two sentences.  

**Answer**: Done 

 ----------------- **Reviewer**: Introduction P. 4: In this sentence, “When considering the subset of peri-urban agricultural systems, these processes are further exacerbated.” I suggest moving the peri-urban fringe definition to the beginning of the paragraph, closer to where this term is introduced.

 Answer: Done -

 ---------------- **Reviewer:** Introduction P. 4: Need to define “soil health” as the definition is not always consistent. It also quickly moves on to the importance of soil health as a whole, but it would help to mention its particular relevance to peri-urban areas, which was the topic of the previous paragraph.

**Answer:** We revised the paragraph by adding a definition of soil health and a sentence that directly mentions its importance to peri-urban areas (page 3).

 ------------------ **Reviewer: **P. 4: “According to 16,..” Should be changed to “author’s name and colleagues” followed by the citation.

**Answer:** Done

 -------------------- **Reviewer: **P. 4: “contest” needs to be changed to “context” in the last paragraph of the introduction.

**Answer:** Done

**Reviewer:** P. 4: The description of the study could be improved in the last paragraph. These “different indicators” need to be explained more, especially if the authors are suggesting they can be used in other studies as indicators of soil health.

**Answer:** We revised the manuscript as suggested by the referee.

 -------------------- **Reviewer: Methods:** The overall organization of the data analysis of the methods is a bit confusing. It makes more sense to break your methods into sequence analysis and statistical analysis, rather than a section for each test.  

**Answer: **We modified and renamed the sections as suggested by the referee.

 ------------------------------- **Reviewer: Methods:** I do not understand the reasoning behind using the “Illumina 16S Metagenomics pipeline” in one section, then, in another section, saying the data was processed again using “QIIME 2.” Only one should be used as they will produce different OTU/ASV tables. No link or reference to the Illumina pipeline was provided, so I was unable to see how the methods compared.

**Answer: **PICRUSt2 requires not only a species abundance table but also the corresponding FASTA file of representative sequences. The output we received from the Illumina 16S Metagenomics workflow consisted of taxonomic abundance summaries and raw FASTQ files, but it did not include a representative-sequence FASTA associated with the OTU/feature identifiers. For this reason, we reprocessed the raw FASTQ files in QIIME 2 to infer ASVs and the corresponding representative sequences FASTA. These QIIME 2 outputs were the inputs used for PICRUSt2. We have revised the Methods to clarify this rationale.

 ---------------------------------- **Reviewer: Methods:** Why not perform beta-diversity analysis? This would be a better comparison of diversity differences across sites as it takes into account the community structure/taxa at each site.

**Answer:** We thank the referee for the suggestion. We have now included a new figure with beta-diversity analysis, a description in the Method section, the results and discussion sections.

 ------------------------- **Reviewer: Methods** The methods need to be checked for tense usage. Often using the present tense when needing to use the past tense when describing tests and sampling.

**Answer:** Done

 ------------------------ **Reviewer: Methods** P. 11: Add a sentence making it clear that the management history is presented in Table 1. I was looking for it in the text because it wasn’t clear that I could find it in the table.

**Answer:** Done

 ------------------------- **Reviewer: Methods** P. 11: Confused on why four quadrants were established and one sampling point was established in each, but only 3 samples were taken.

**Answer: **  The referee is right, the method was not clear. We analysed three samples randomly for each point. We corrected this point now at page 8.  

 ------------------------------------ **Reviewer: Methods** P. 11: When were the samples collected? Include the year and the month at least. This is important since it is said some land uses were established in 2019, so it is helpful to know how long since the establishment of the site where samples were taken.

**Answer: ** We have now added this information in  Study Area of Methods section (page 8).

 -------------------------------- **Reviewer: **Methods P. 14: There is no information given on how soil parameters were measured. Either cite other papers that have this information, or, if they were sent somewhere, include the location and the link so readers can find the methods there.

**Answer:** We have now included this information in the Methods’ section (page 8).

 -------------------------- **Reviewer: **Methods P. 14: Change “DNA quality control checks” to “DNA quality and quantity were checked” since the Nanodrop is generally for quality and Qubit for quantity.

**Answer:** Done

 --------------------- **Reviewer:** Methods P. 14: Add the specific 16S primers (e.g., 314F and 806R).

**Answer:** Done.

 ------------------ **Reviewer:** Methods P. 14: Add a link or citation where the specific PCR protocol can be found. I also recommended including a short summary, such as whether it was done in replicates, in two steps, etc.

**Answer:** Done

 ------------------- **Reviewer:** Methods P. 14: Add a link or citation for the “16S Metagenomic pipeline”. Was there a different pipeline for the ITS, or was it adapted at all for differences between ITS and 16S reads?

**Answer:** Done -

 ------------------- **Reviewer: **Method P. 14: The database versions used are dated. Ideally, should use more recent releases of both databases.

**Answer:** The amplicon sequencing data were analyzed using the 16S Metagenomics application available in the Illumina BaseSpace Sequence Hub. This pipeline performs read processing and taxonomic classification using a k-mer–based Naïve Bayesian classifier derived from the Ribosomal Database Project (RDP) algorithm (Wang et al., 2007). The taxonomic assignment for 16S rRNA sequences was performed using the RefSeq RDP 16S v3 database. For the ITS datasets, the same analytical framework was used; however, taxonomic assignment was performed using the UNITE reference database, which is specifically curated for fungal ITS sequences (Kõljalg et al., 2013). Therefore, the pipeline itself was not fundamentally different, but the reference database used for taxonomic classification was adapted to account for the differences between bacterial 16S and fungal ITS markers. We now added this part in the Methods section.

 --------------------- **Reviewer:** Methods P. 14: It is mentioned that negative controls were included. Were any sequences found in the negative controls, and if so, include how they were accounted for in the data.

**Answer:** The negative control included during library preparation did not generate a detectable library profile. During the sequencing run no reads were obtained from the negative control sample. Therefore, no sequences from the negative control were present or required filtering in the downstream analyses. We now added this part in the Methods section.

 -------------------- **Reviewer:** Methods P. 14: While using relative abundance normalization for the data is fine, information should be included on the average sequencing depth for each dataset, and if there was a cutoff on the minimum number of sequences considered usable for a sample. Given soil diversity, sequencing depth can be important.

**Answer: **The average sequencing depth across samples was approximately 40,000 reads per sample. For quality control purposes, we considered ~10,000 reads per sample as the minimum threshold required for a sample to be included in the downstream analyses. This threshold was selected to ensure sufficient sequencing depth for reliable microbial community profiling, while minimizing potential biases associated with low-coverage samples. Similar thresholds have been widely used in microbiome studies to ensure adequate representation of microbial diversity and to improve the robustness of comparative analyses.  

 -------------------- **Reviewer:** Methods P. 15: It should be mentioned that the metrics used are both alpha-diversity metrics.

**Answer**: Done

 --------------------- **Reviewer: **P. 15: Does the “16S Metagenomic pipeline” produce OTUs or ASVs? It is generally just referred to as species-level assignments in the methods.

**Answer:** The Illumina BaseSpace 16S Metagenomics pipeline does not generate OTUs or ASVs. Instead, it performs direct taxonomic classification of reads using a k-mer–based Naïve Bayesian classifier derived from the Ribosomal Database Project (RDP) algorithm. Each read is assigned directly to the most probable taxonomic level based on the reference database, and the output is therefore reported as taxonomic profiles rather than OTU or ASV tables.

 ------------------- **Reviewer:** Methods P. 16: For the UpSet plots, was a taxa considered to be present in both land uses if it was present in just one of the replicates or more?

**Answer**:  We agree with the referee and we have now clarified this point in the manuscript in the Methods section (Statistical Analysis on page 10).

 ------------------------ **Reviewer:** Methods P. 16: Was the data checked for normality and homogeneity of variance before determining that a parametric test (ANOVA) was the right choice? If so, include that in the methods.

**Answer**:  We checked normality across land-uses applying the Shapiro–Wilk test and homogeneity of variance using Levene’s test. However, due to the small number of replicates, we acknowledge that the results of these tests should be interpreted cautiously. We included these details in the Methods section.

 ------------------------ **Reviewer:** Methods P. 18: Was there a correlation coefficient cutoff value to be considered “strongly” correlated?

**Answer**:  We did not apply a correlation coefficient threshold.  “Strongest” correlations were defined operationally for visualization purposes as the **top 20 variable pairs ranked by absolute Pearson’s r** within each land-use type. This filtering was used to reduce visual clutter and highlight the most important associations in the heatmap, rather than to assert a clear definition of  “strong” correlation.

 ---------------------- **Reviewer:** Methods P. 19: Did you consider the correlation between the abiotic factors when doing your RDA? Also, need to add the vectors of the abiotic factors to the RDA so readers can see which factors were associated with each land-use type.

**Answer:** We have now assessed collinearity among the abiotic predictors and repeated the RDA after removing redundant variables using a pairwise-correlation threshold (|r| = 0.9), retaining only predictors below this threshold. The resulting ordination was highly similar to the original analysis, indicating that our conclusions are robust to predictor multicollinearity. We have now added this procedure to the Methods section. We also added vectors of the retained abiotic parameters to the RDA plots.

 ---------------------------- **Reviewer: **Methods P. 19: Please add a statement about the use of PICRUSt, such as, “however, these predictions are based on reference genomes and do not represent direct measurements of functional genes. Therefore, results should be interpreted cautiously,” considering its limitations.

**Answer:** Done

 --------------------- **Reviewer: **Results For the results, my main comments would be to reduce the number of figures to make the message more concise. For example, the alpha-diversity metrics are shown by field with box plots (Fig. 2), then by replicate (Fig. 3). Unless a main point is within site variation, then Figure 3 is not needed. The same for showing heatmaps of the top 20 correlations between abiotic factors, then showing heatmaps with all variables.

**Answer: **We agree with the referee and we have now moved Fig.3 in the supplementary together with Fig.9, 10, 11 and 12 **.**

 ---------------------- **Reviewer: **Results In the results text, there needs to be a stronger focus on the differences between land uses to convey that converting to certain land-use types may improve “soil health,” as seen by increases in diversity, richness, OM, etc., to match the story set out in the introduction.

**Answer:** We revised the Results section as suggested by the referee.

 ----------------------- **Reviewer: **Results P. 4: When saying “significantly” in terms of results, it can be helpful to put the p-value in parentheses after it, especially since the method is after the results, where significance is defined. I recommended this for the whole results section. **Answer:** Done

 ------------------------ **Reviewer: **Results P. 4: “certain genera significantly more abundant in specific samples and in specific land-use types”, which ones especially on a land-use base comparisons?

**Answer: **We expanded those sentences by clearly specifying some of the observed patterns.

 ---------------------- **Reviewer: **Results P. 4: The genera “Mortierella, Fusarium, and Trebouxia” need to be italicized.

**Answer:** Done -

 ----------------------- **Reviewer: **Results P. 4: I do not think this conclusion is supported because no differences in relative abundance were explained between sites in the text and no differences in organic inputs have been presented yet, “From an agroecological point of view, this results clearly demonstrate the role of agronomical management that enhance soil organic inputs (e.g., manure, green mulch, compost application, agroforestry litterfall) in sustaining fungal diversity.”

**Answer:** We have revised this statement to avoid implying a mechanistic explanation and we moderate our statement by substituting the expression ‘this results clearly demonstrate…’  with ‘the results are consistent with the idea that…’

 ------------------------------- **Reviewer: **Results P. 4-5: For the differences in alpha-diversity metrics, state if they were significant, not just noticeable. The statement about replicating following it makes it sound like you would have found significant values if you had more replicates, which is not necessarily true. Consider rewording or removing that statement.

**Answer:** We agree. We have revised the text to explicitly state which alpha-diversity comparisons were statistically significant and removed wording regarding replication-size. We now describe the observed patterns descriptively and report significance only when supported by the statistical tests.

 ---------------------------- **Reviewer: **Results P. 5: Fungi is a kingdom, not a domain.

**Answer:**  Fixed

 ------------------------ **Reviewer: **Results P. 5-6: “Such diversification contributes to ecosystem resilience and multifunctionality, which are essential for effective biological pest control.” A statement like this is better for the discussion, so you can show that other studies have shown this. Otherwise, it appears to be an unsupported claim in the results.

**Answer:** This sentence has been removed.

 --------------------------- **Reviewer: **Results P. 6: Again, explain the differences in what taxa you saw that were significantly different between land uses. The figure captions also say genera instead of phyla. It would also make more sense to talk about the differences in phyla (Fig. 4/5) before talking about the genera (Fig. 1).

**Answer:** We corrected accordingly

 ------------------------------ **Reviewer: **Results P. 6: What is meant by “bacterial and fungal profiles”? Do you mean the special-level abundance tables? This information on what went into the tables needs to be included in the Figure 6 caption. Based on the coefficient gradient for your heatmap, I also cannot tell if it includes negative correlations. I number at the bottom of the gradient needs to be included.

**Answer:** We corrected accordingly.

 -------------------------- **Reviewer: **Results P. 7: The conclusions on soil stability based on abiotic variable correlation need to be moved to the discussion, so examples that support this can be cited. It would also be a good place to discuss how the management specific to the crop and reforested sites can lead to shared drivers of these properties.

**Answer:** We corrected accordingly.

 ------------------- **Reviewer: **Results P. 8: Redundant/highly correlated variables can impact a PCA and RDA. It would be useful to include details in the figures' captions and in the methods section on how this was handled, since strong correlations between factors were shown in Figure 7.

**Answer:** We corrected accordingly

 ---------------------- **Reviewer: **Results P. 8: Discussing the differences in abiotic factors between land uses (Fig. 13), which help show what factors shift under the different management and may help support the PCA results below, showing that there are differences in soil factors by land use.

**Answer: **We corrected accordingly.

 ----------------------- **Reviewer: **Results P. 8: I believe I mentioned this before, but all the abiotic vectors are in the RDA, so you can discuss which factors were associated with different land uses.

**Answer:** We have now described the associations between vectors of explanatory abiotics and different land-uses in the Results section where RDA is discussed.

 -------------------- **Reviewer: **Results P. 9: For the functional differences, again explain the important differences between land use. Did you see a loss or gain of a certain function with land use?

**Answer:** We added a paragraph describing some examples of functionalities that were comparatively more present in a land-use compared to the others, for all three categories of functions.

 ------------------- **Review **Discussion Overall, the discussion needs to focus more on how the results presented were caused by differences in land use and how the management practices associated with those land uses may have shaped them. This then needs to be brought back to the idea of soil health. Currently, it is not focusing enough on the differences between land uses, and again, it just says there were broad differences between land uses.

**Answer: **We have substantially revised the Discussion to highlight land-use contrasts and to interpret observed patterns in terms of land-use–associated management contexts. Moreover, we moved interpretive statements (e.g., implications of abiotic correlation structure and diversification benefits) from Results to Discussion, as suggested. Relevant citations have been added in support of interpretations regarding diversification, ecosystem multifunctionality, and pest-suppressive agroecosystems.

**----------------------** **Review **Discussion Altogether, the authors present an interesting study on how land use type may influence soil microbial diversity and overall soil health in a peri-urban landscape. However, significant revisions are needed to improve the clarity, robustness, and alignment between the stated objectives and the results. The authors should provide more detail in the Materials and Methods section on sequencing data and management practices for each land-use type. The number of figures should be reduced, and the order of figures reconsidered to group similar topics (e.g., keep all relative abundance differences together). The results and discussion should focus more on differences between land-use types, mentioning specific differences and how management practices influence soil properties and microbial communities to support claims about soil health. Lastly, moderate claims about mechanisms and resilience to better align with the data presented.

**Answer: **We thank the reviewer for the very useful and detailed comments. We have now extensively revised the manuscript incorporating all the suggestions.

# Reviewer report: Reviewer response for version 1

**DOI:** 10.21956/openreseurope.23877.r69039

**Published:** 2026-02-24

**Authors:** Shefali Azad

**Affiliations:** 1 Archbold Biological Station, Lake Placid, Florida, USA

This study is conceptually strong and analytically rich and tackles a timely ecological issue. That is, the relationships between microbial diversity, soil health, and land use, which ultimately informs the ecological trade-offs of land management. 

 The methods section was very detailed and allows for replication by other parties. Is it a decision of the journal to have results before methods? I was confused by this layout, but it did create an interesting reading/interpreting experience. The relatively low #replicates does complicate the interpretation of statistical results, given that soil microbial communities are patchy on a finer scale - I was glad to see that this was addressed at the end of the discussion, but perhaps it could be shifted to earlier in the discussion to add context. In general - what are the results saying about beta diversity of the land use types, considering sample size? 

 I appreciated the variety of statistical analysis and visualizations. In particular I think the UpSet diagram could be better explained - I had not encountered this before and it revealed a lot of information, but it took external searching to understand it. 

 Minor note, this line seemed incomplete: "Agroforestry soils were characterized by the highest number of unique taxa, whereas reforested soils showed the lowest richness and diversity, aligning with previous studies that highlight the early successional stage of reforestation systems" - aligning with previous studies that highlight what about the early successional stage?

 This line: "The predominance of Mortierella, Fusarium, and Trebouxia in fungal communities, as well as the dominance of Acidobacteria and Gemmatimonadetes in bacterial communities, underscores the potential of these taxa as bioindicators of soil health and management intensity." - is not necessarily a leap, but perhaps a functional extrapolation? Does presence signal relative soil health if widespread across land-use types, and through what mechanisms? (Mortierella unpacking excluded).

Is the study design appropriate and does the work have academic merit?

Yes

Is the work clearly and accurately presented and does it cite the current literature?

Yes

If applicable, is the statistical analysis and its interpretation appropriate?

Yes

Are all the source data underlying the results available to ensure full reproducibility?

Yes

Are the conclusions drawn adequately supported by the results?

Yes

Are sufficient details of methods and analysis provided to allow replication by others?

Yes

Reviewer Expertise:

Agroecology, biotechnology, data management, statistical analysis (wildlife biology focus).

I confirm that I have read this submission and believe that I have an appropriate level of expertise to confirm that it is of an acceptable scientific standard.

# Response

**Published:** 2026-03-25

**Authors:** Caterina La Porta

**Affiliations:** Universita degli Studi di Milano, Milan, Lombardy, Italy

**REVIEWER #1**

**Reviewer:** This study is conceptually strong and analytically rich and tackles a timely ecological issue. That is, the relationships between microbial diversity, soil health, and land use, which ultimately informs the ecological trade-offs of land management. 

**Answer:** We sincerely thank the reviewer for the careful and thoughtful evaluation of our manuscript and for recognizing its conceptual strength and analytical richness. We address each comment below and describe the revisions implemented in the updated manuscript. --

**Reviewer:** The methods section was very detailed and allows for replication by other parties. Is it a decision of the journal to have results before methods? I was confused by this layout, but it did create an interesting reading/interpreting experience.

**Answer: **We thank the reviewer for highlighting this point. The structure follows the Open Research Europe article template, which allows flexibility in manuscript organization. In our case, we adopted a Results-first structure to foreground the empirical findings before detailing methodological procedures.

**--** **Reviewer**: The relatively low #replicates does complicate the interpretation of statistical results, given that soil microbial communities are patchy on a finer scale - I was glad to see that this was addressed at the end of the discussion, but perhaps it could be shifted to earlier in the discussion to add context.

**Answer:** We fully agree that soil microbial communities exhibit fine-scale spatial heterogeneity, and the limited number of biological replicates constrains statistical power. In the revised manuscript, we have moved the discussion of replication limits earlier in the Discussion section on page 19, accordingly.

**--** **Reviewer #1**: In general - what are the results saying about beta diversity of the land use types, considering sample size? 

**Answer: **We thank the referee for the suggestion. We have now included beta-analysis and added the description in the Methods, Results and Discussion sections

 ---- **Reviewer**: I appreciated the variety of statistical analysis and visualizations. In particular I think the UpSet diagram could be better explained - I had not encountered this before and it revealed a lot of information, but it took external searching to understand it. 

**Answer:** We have added a brief explanation to the Methods section of the manuscript describing how to interpret the UpSet diagram (set sizes, intersection sizes, and how intersections correspond to shared vs. land-use–specific taxa) on page 9.  

 --- **Reviewer:** Minor note, this line seemed incomplete: "Agroforestry soils were characterized by the highest number of unique taxa, whereas reforested soils showed the lowest richness and diversity, aligning with previous studies that highlight the early successional stage of reforestation systems" - aligning with previous studies that highlight what about the early successional stage?

**Answer:** Our intent was to indicate that the comparatively lower richness and diversity in reforested soils is consistent with previous studies reporting that early successional reforestation systems often host simpler, less diverse microbial communities. We have revised the sentence accordingly on page 13.

**----** **Reviewer #1**: This line: "The predominance of Mortierella, Fusarium, and Trebouxia in fungal communities, as well as the dominance of Acidobacteria and Gemmatimonadetes in bacterial communities, underscores the potential of these taxa as bioindicators of soil health and management intensity." - is not necessarily a leap, but perhaps a functional extrapolation? Does presence signal relative soil health if widespread across land-use types, and through what mechanisms? (Mortierella unpacking excluded).

**Answer:** We agree with the referee and we have now changed the sentence accordingly on page 13.
